# An antifungal effector from a plant-parasitic nematode modulates host fungal community composition and supports ecological fitness

**DOI:** 10.1038/s41522-026-00954-4

**Published:** 2026-03-10

**Authors:** Dong-Zhen Li, Yongxia Li, Xuan Wang, Yuqian Feng, Yuhang Liu, Can Yang, Wei Zhang, Xiaojian Wen, Zhenkai Liu, Wandong Yin, Xingyao Zhang

**Affiliations:** 1 Key Laboratory of Forest Protection of National Forestry and Grassland Administration, Ecology and Nature Conservation Institute, Chinese Academy of Forestry, Beijing, China; 2 Co-Innovation Center for Sustainable Forestry in Southern China, Nanjing Forestry University, Nanjing, China

**Keywords:** Ecology, Ecology, Microbiology

## Abstract

Host-associated microbiomes are increasingly recognized as key determinants of plant health, disease development, and ecosystem functioning. Plant pathogens, especially fungal pathogens, have been reported to secrete antimicrobial effectors to modulate the host microbiota and promote colonization. Plant-parasitic nematodes (PPNs) could also modulate host microbial communities, but the processes involved remain to be clarified. Here, we identify a secreted antifungal effector, BxylTLP6, from *Bursaphelenchus xylophilus*, the causal agent of pine wilt disease. BxylTLP6 degrades fungal cell walls and inhibits multiple plant-associated fungi, while the released oligoglucans serve as food-derived cues that guide nematode foraging toward fungal resources. In planta, silencing *Bxyltlp6* significantly delayed disease progression. ITS-based mycobiome profiling revealed that BxylTLP6 modulates the pine endophytic fungal community by promoting Ascomycota, suppressing Basidiomycota, inhibiting wood-decaying fungi, and enriching pathogenic or parasitic taxa. These shifts are associated with enhanced nematode survival and pathogenicity. Our findings support the view that a TLP effector can modulate behavior and influence the host fungal microbiome, shedding light on how PPN may manipulate microbial environments to enhance their fitness.

## Introduction

Plant disease development is increasingly recognized as the outcome of multilayered interactions among hosts, pathogens, and their associated microbiomes^1^. Beyond the classical view that pathogenic success is determined by direct host-pathogen interactions^2^, a growing body of evidence demonstrates that pathogens can manipulate host-associated microbial communities to create a favorable ecological niche^3–5^. Recent studies further reveal that several fungal and oomycete pathogens secrete antimicrobial effectors capable of directly reshaping the host microbiota, thereby enhancing their ability to colonize and cause disease^6–12^. In addition to such direct antimicrobial activities, pathogens can also manipulate their host immune responses and metabolic states, thereby indirectly restructuring host-associated microbial communities to generate a more permissive niche for infection^13–15^. These observations underscore the importance of microbiome manipulation as a widespread pathogenic strategy.

Plant-parasitic nematodes (PPNs) are major plant pathogens that inflict substantial damage through mechanical penetration and nutrient extraction^16^. During infection, PPNs encounter strong selective pressures from host-associated symbiotic microorganisms that can directly inhibit nematode development, produce anti-nematode metabolites, or modulate host immunity in ways that restrict nematode colonization^17,18^. Numerous studies have shown that PPNs invasion is accompanied by marked shifts in plant-associated microbial communities^18–21^. The host-driven mechanisms underlying these microbiome changes, including metabolic reprogramming and immune signaling, are increasingly well understood^22–24^. However, whether PPNs can secrete antimicrobial effectors to modulate the host microbiota, analogous to fungal pathogens, remains insufficiently explored. Emerging evidence provides initial support for this possibility. The root-knot nematode *Meloidogyne incognita* effector MiMIF-2 modulates the rhizosphere microbiome when expressed in transgenic *Arabidopsis*^25^, and the soybean cyst nematode *Heterodera glycines* secretes the chitinase HgCht2 that hydrolyzes lipochitooligosaccharide signals required for rhizobial and mycorrhizal symbioses, thereby disrupting beneficial plant-microbe associations^26^. These findings suggest that PPNs may employ specific molecular tools to influence the host microbiome. Despite these advances, our understanding of how PPNs interact with and potentially modulate host-associated symbiotic microorganisms remains limited, and the molecular strategies underlying nematode-driven microbiome remodeling are still largely unexplored.

*Bursaphelenchus xylophilus*, the pine wood nematode (PWN), is a globally quarantined pest and the causal agent of pine wilt disease, the most destructive forest disease in East Asia and an emerging threat to European forests^27–31^. Unlike sedentary plant-parasitic nematodes such as root-knot and cyst nematodes, which induce specialized feeding structures and remain fixed at permanent feeding sites^32,33^, PWN is migratory and moves extensively within the xylem^34,35^. Its survival, reproduction, and pathogenicity are strongly shaped by interactions with the diverse endophytic fungi inhabiting pine tissues. PWN exhibits a facultative dual feeding strategy, feeding on plant parenchyma cells while the host is alive (phytophagous phase) and shifting to fungal feeding as tissues decline (mycophagous phase)^35–37^, which enables it to utilize a broad range of pine-associated fungi, including common endophytes (e.g., *Botrytis cinerea*, *Cladosporium herbarum*) and pathogenic species (e.g., *Sirococcus conigenus*, *Sphaeropsis sapinea*)^38,39^. Some fungi isolated from healthy or dead pine trees can suppress pine wood nematode populations in both in vitro and in vivo bioassays, including species of *Aureobasidium*, *Aspergillus*, *Cephalosporium*, *Fusarium*, *Gliocladium*, *Mortierella*, *Rhizoctonia*, *Trichoderma*, and *Verticillium*^38,40–44^. The nematophagous fungi such as *Esteya vermicola* and *E. floridanum* can infect and kill PWN directly^45,46^, while wood-decay fungi colonizing dead trees can also suppress nematode populations^47,48^. Conversely, some fungi form mutualistic associations with PWN, such as *Sporothrix* sp. 1 in China, which boosts nematode fecundity and spread while benefiting from nematode-mediated dispersal^49^. Therefore, during its invasion and colonization of the host, the PWN engages in complex interactions with the microorganisms within its ecological niche.

Omics studies further reveal that PWN infection markedly modulates the host mycobiome. Across multiple pine species, infection typically reduces fungal richness and diversity in needles and endophytic compartments, while enriching pathogenic and saprotrophic Ascomycota, such as Ophiostomatales (e.g., *Ophiostoma*, *Graphilbum*), *Diplodia*, and various yeast-like fungi. In contrast, plant-beneficial or potentially antagonistic fungi (e.g., *Trichoderma, Penicillium, Russula* and phyllosphere yeasts such as *Aureobasidium*) are more abundant in healthy trees^38,43,50–59^. Together, these findings illustrate the complex and dynamic fungal environment that shapes PWN fitness and disease progression and underscore PWN as an excellent system for dissecting nematode interactions with host microbiomes and their molecular mechanisms.

Considering the extensive interactions between PWN and diverse fungal taxa, we focused on thaumatin-like proteins (TLPs), a conserved family of antifungal proteins present in plants, insects, fungi, and nematodes^60,61^. TLPs, named for their structural resemblance to the sweet-tasting protein thaumatin, possess a conserved β-1,3-glucanase-like fold and are classified within the GH64-TLP superfamily^62,63^. Their defining structural features include the highly conserved thaumatin signature motif and the REDDD motif, together with 10 or 16 cysteine residues forming 5 or 8 disulfide bonds, respectively. TLPs comprise three domains: domain I, an 11-strand β-barrel forming the protein core; domain II, containing an α-helix and disulfide-rich loops; and domain III, featuring a β-hairpin and coil motif. A cleft between domains I and II is thought to be the key structural element underlying their antifungal activity^64,65^. These proteins have been widely reported in plants, where they exhibit β-1,3-glucanase activity and exert antifungal effects by degrading fungal cell walls^66–68^. In insects, TLP genes have been acquired via horizontal gene transfer (HGT) into whitefly species such as *Bemisia tabaci* and *Trialeurodes vaporariorum*, where they function to either resist entomopathogenic fungal infection or suppress host plant immune responses^69^. These studies indicate a potential role for TLPs in the interactions between PWN and associated fungal communities. Despite their broad presence, TLPs remain largely unstudied in nematodes. Therefore, this study aims to determine whether the TLPs secreted by PWN function as effectors that mediate the nematode’s interactions with host-associated symbiotic microbial communities.

In this study, we identified a secreted TLP gene, *BxylTLP6*, from the pine wood nematode (PWN) and characterized its structural, functional, and ecological roles. BxylTLP6 exhibits β-1,3-glucanase activity and inhibits multiple pine-associated fungi. It mediates nematode foraging behavior by degrading fungal cell walls. Meanwhile, this antifungal activity influences the structure and function of the host-associated symbiotic fungal community, which is likely to contribute to nematode fitness and virulence during colonization. These insights advance our understanding of the capacity of PPNs to influence host-associated microbial communities through specialized molecular mechanisms.

## Methods

### Nematode culture

The PWN population and the fungus *Botrytis cinerea* used in this study were originally isolated in October 2012 from naturally infected *Pinus massoniana* in Ningbo, Zhejiang Province, China. Nematodes were extracted using the Baermann funnel method and maintained long-term on corn-based medium inoculated with *B. cinerea* at 4 °C, with subculturing every three months. Dried corn kernels (240 g) were washed, air-dried, and soaked in an equal volume of sterile water, then boiled for 10 min until fully hydrated. About 40 g of the boiled kernels were transferred into 100-mL Erlenmeyer flasks and sterilized at 121 °C for 2 h. After cooling, each flask was inoculated with 6-7 uniform plugs of *B. cinerea* from PDA plates and incubated at 25 °C in the dark for 14 days until the kernels were fully colonized. The PWN and fungus strain is preserved at the Institute of Ecology and Nature Conservation, Chinese Academy of Forestry.

Prior to experiments, nematodes were rinsed from corn-based cultures into 15 mL centrifuge tubes with sterile water, centrifuged at 12,000 rpm for 3 min, and the supernatant discarded. The nematodes were transferred onto PDA plates overgrown with *B. cinerea* mycelia and incubated at 25 °C for 7 days for activation. Activated nematodes were collected using the same procedure.

### Identification and phylogenetic analysis of TLP genes

We analyzed 160 nematode genomes, including 159 species from WormBase (https://parasite.wormbase.org/species.html) and *B. mucronatus* from NCBI (GCA_025436335.1). The genomic information of the species is provided in Supplementary Data 1. For each genome, all annotated protein sequences (primary transcripts) in FASTA format and gene annotation files were downloaded. Amino acid sequences of five *Caenorhabditis elegans* TLP genes from UniProt were used as queries for BLAST searches against each genome in TBtools-II (*E*-value < 1e−5)^70^. Sequences with <30% identity were discarded. Remaining hits were screened using the NCBI Conserved Domain Database; non-TLP family proteins were excluded. Signal peptides were predicted with SignalP 5.0, and sequences lacking signal peptides were removed. DNAMAN v6 was used for alignment; sequences lacking the conserved “REDD” motif or incomplete ORFs were discarded. In total, 203 full-length TLP ORFs were identified from 58 nematode species (Supplementary Data 2). In addition, we retrieved 22 TLP protein sequences from *Arabidopsis thaliana*, 9 fungal TLP sequences, and 20 insect TLP sequences from UniProt (https://www.uniprot.org/), as well as 11 TLP sequences from *P. massoniana* obtained from previously published transcriptomes^71^. The isoelectric point and molecular weight of the proteins were calculated using the ExPASy tool (https://web.expasy.org/compute_pi/). TLP protein 3D structures were predicted with AlphaFold Serverand secondary structures compared with PDB references using ESPript 3 (https://espript.ibcp.fr/ESPript/ESPript/index.php). All these sequences contain complete conserved domains and signal peptides (Supplementary Data 2). Multiple sequence alignment was performed with ClustalW2, and phylogenetic trees were constructed in MEGA 7.0 using the neighbor-joining method. Node support values were estimated using 10000 ultrafast bootstrap replicates. Trees were visualized and annotated with iTOL (http://itol.embl.de). Nematode species were classified by feeding strategy (plant, fungi, bacteria, insect, or small invertebrates) based on literature sources (Supplementary Data 3).

### Statistical analysis of the association between TLP copy number and habitat microbial diversity

To compare the ecological microbial pressures experienced by different nematodes, we assigned each species to a microbial-diversity category (High, Medium, Low) based on microbiome characteristics of their habitats (Supplementary Data 3). Soil and decaying plant material substrates were classified as High microbial diversity due to their exceptionally rich, heterogeneous microbial communities documented in global soil microbiome surveys and detritus-based food webs^72,73^. Plant associated, insect-associated, and extreme soils were categorized as Medium diversity because they harbor moderate microbial richness but are shaped by strong plant- or insect-specific selective pressures^3,74,75^. Vertebrate-associated environments, deep rock-fracture water systems, and deep marine sediments were categorized as Low diversity due to their reduced microbial richness and strong abiotic or host-driven filtering^76–78^.

To test whether TLP gene copy number is associated with habitat microbial diversity, we calculated Spearman’s rank correlation between TLP copy number and the diversity class. We further applied a non-parametric Kruskal-Wallis test to compare TLP copy number among the three diversity classes, followed by Dunn’s post hoc test for pairwise comparisons. In addition, we fitted a simple linear regression model with TLP copy number as the response and microbial-diversity class as the predictor to visualize the overall trend.

### β-1,3-glucanase activity assay

Two *tlp* genes from PWN, designated *Bxyltlp5* and *Bxyltlp6*, were amplified using the primers listed in Supplementary Table 1. The predicted isoelectric point and molecular weight of BxylTLP5 were 8.09 and 27.4 kDa, respectively, whereas those of BxylTLP6 were 8.38 and 30.3 kDa. Recombinant TLP proteins were produced via prokaryotic expression using the NEBExpress^®^ MBP Fusion and Purification System (New England Biolabs, Beijing, China). The pMAL-c6T vector provided in the kit, which contains both 6×His (Histidine) and maltose-binding protein (MBP) tags, was used for expression, and TEV protease was employed to remove the tags. The purified recombinant proteins were dissolved in phosphate-buffered saline (PBS, pH = 7.4) and verified by SDS-PAGE.

Purified proteins were diluted to 1 mg/mL in PBS prior to β-1,3-glucanase activity measurement. β-1,3-Glucanase activity was determined using a β-1,3-Glucanase Activity Assay Kit (Boxbio, Beijing, China). The assay was performed by incubating the enzyme sample with laminarin substrate at 37 °C for 60 min. The reaction was terminated by boiling for 5 min, followed by the addition of DNS color reagent and a second boiling step to develop the chromogenic product. After cooling to room temperature, absorbance was recorded at 540 nm using a visible-light spectrophotometer. A glucose standard curve (0.2–1.0 mg/mL) was generated to quantify the amount of reducing sugars released. Enzyme activity was calculated based on the difference in absorbance between the reaction mixture and its corresponding control. One unit of β-1,3-glucanase activity was defined as the amount of enzyme required to release 1 mg of reducing sugar per hour. Because the enzyme samples were prepared as 1 mg/mL purified protein solutions, activity was expressed directly as U per mg protein. Each protein assay included five technical replicates.

### Inhibition of fungal growth by TLPs

Antifungal activity assays were performed by placing protein-soaked sterile filter paper discs onto PDA plates pre-inoculated with the indicated fungal strains. Antifungal activity assays were conducted using *B. cinerea* and nine additional fungal isolates obtained from *Pinus* tissues, including *Ophiostoma ips* (FXY693, FXY10, FXY619, FXY789, FXY778), *Botryosphaeria dothidea* (FXY94), *Irpex lacteus* (FXY528, FXY618), and *Phanerochaete concrescens* (FXY475). To specifically assess the antifungal activity of BxylTLPs, fungi strains were cultured on PDA plates. For each assay, a 5-mm mycelial plug was placed at the plate center, and four sterile paper discs (6 mm diameter) were positioned equidistantly around the inoculum. Each disc was loaded with 50 µL of either heat-inactivated BxylTLP proteins or purified active BxylTLP proteins solution (15 mg/mL). Plates were incubated at 25 °C, and fungal growth inhibition was evaluated by observing and comparing the growth patterns around the discs. Antifungal activity assays were repeated five times independently.

### Expression analysis of Bxyltlp genes at different developmental stages

Nematodes were rinsed from *B. cinerea* cultures into 9 cm glass petri dishes with ddH_2_O. After 30 min, eggs adhered to the dish bottom were retained, and other nematodes were removed. Eggs were incubated in 10 mL ddH_2_O at 25 °C for ~30 h to obtain second-stage juveniles (L2). L2 were transferred to *B. cinerea* PDA plates, yielding L3 after 24 h and L4 after 52 h. Adults were obtained by separating late L4 by genital morphology and culturing for an additional 24 h. Nematodes at each stage were flash-frozen in liquid nitrogen and stored at –80 °C. Total RNA was extracted by RNeasy Plus Mini Kit (QIAGEN, Hilden, Germany), reverse transcribed by HiScript IV RT SuperMix (Vazyme, Nanjing, China). RT-qPCR was performed using the SupRealQ Ultra Hunter SYBR qPCR Master Mix (U+) (Vazyme) with the primers in Supplementary Table 1. The β-actin gene (GenBank accession number EU100952.1) was used as the internal reference gene for analyzing PWN gene expression^79,80^. RT-qPCR assays were performed on a LightCycler^®^ 480 system (Roche, Basel, Switzerland). Relative expression was calculated by the 2^−ΔΔCt^ method. Each stage had five biological replicates.

Two- to three-year-old *P. massoniana* seedlings of similar vigor were selected. A downward-angled (45°) hole was drilled into the main stem 10–15 cm above the soil line, and 50 µL of suspension containing 2000 mixed-stage nematodes was injected. The wound was sealed with parafilm. Seedlings were watered weekly, and symptoms were recorded. For temporal gene expression analysis, 2 cm stem segments 3 cm above the inoculation site were collected at 1, 7, 15, and 30 days post-inoculation (dpi). Bark was removed, xylem ground in liquid nitrogen, and RNA extracted for RT-qPCR as described. Each time point had five biological replicates, each consisting of three seedlings.

### In situ hybridization

mRNA in situ hybridization (ISH) was performed following previously described protocols^81,82^. Unlike Globodera pallida, the pine wood nematode does not need to be fragmented; intact nematodes can be used directly for permeabilization and hybridization. DIG-labeled antisense and sense probes were generated by asymmetric PCR (primer sequences in Supplementary Table 1) using the DIG Northern Starter Kit (Roche Diagnostics, Mannheim, Germany). Hybridization and signal detection were conducted with the DIG-High Prime DNA Labeling and Detection Starter Kit II (Roche Diagnostics). Samples were examined using a Zeiss Axio Image M2 microscope (Zeiss, Oberkochen, Germany). Whole-mount ISH was performed on L4 larvae and adult nematodes cultured on *B. cinerea*, as these stages exhibit relatively high expression of BxylTLP5 and BxylTLP6 and provide optimal morphology for microscopic observation.

### Feeding preference assays

To assess PWN feeding preference, stem segments (~1 cm diameter, ~2.5 cm length) were excised from wilted *P. massoniana* seedlings and sterilized by autoclaving. A 5 mm longitudinal through-hole was drilled, and both ends were cut at an oblique angle. An 8 mm agar plug bearing fresh *B. cinerea* mycelia was placed at one end. A fresh healthy *P. massoniana* stem segment (~1 cm diameter, ~2 cm length, oblique cut) was attached to the opposite end. A suspension containing 5000 mixed-stage nematodes in 200 µL sterile water was introduced into the through-hole. Assemblies were sealed with parafilm and incubated at 25 °C in darkness for 24 h. The fungal plug and pine segment were then separately chopped, immersed in distilled water for 2 h, and nematodes were collected and counted. Fifteen biological replicates were performed.

To assess PWN feeding preference within-tissue choice assay, healthy *P. massoniana* stem segments (~1 cm diameter, 6 cm length) were drilled at each end (3 mm diameter, ~2 cm deep, longitudinal) and at the midpoint (3 mm diameter, ~5 mm deep, perpendicular). *B. cinerea* mycelia were scraped from PDA plates into centrifuge tubes and briefly disrupted using an ultrasonic cell disruptor until the hyphae were evenly dispersed. The suspension was centrifuged at 5000 rpm for 10 min, the supernatant was discarded, and the hyphal pellet was washed three times with sterile water. PBS alone (100 µL) was added to one end hole (control), and an equal volume of fungal suspension to the opposite hole. The midpoint hole received 2000 nematodes in 30 µL sterile water. Holes were sealed with parafilm and incubated at 25 °C in darkness for 3 days. Two-centimeter segments from each end were chopped, soaked in distilled water for 2 h, and nematodes counted. The same procedure was repeated with diseased and dead pine stems. Fifteen biological replicates were conducted per condition.

### RNA interference (RNAi)

Double-stranded RNA (dsRNA) targeting *Bxytlp5*, *Bxytlp6* and green fluorescent protein (GFP) gene was synthesized using the T7 RiboMAX^TM^ Express RNAi System (Promega, Madison, USA) with the primers in Supplementary Table 1. Concentration and purity were measured by NanoDrop OneC (ThermoFisher Scientific, Waltham, USA), and integrity verified by 1.2% agarose gel electrophoresis. L3 larvae (~20,000) were soaked for 36 h at 25 °C in the dark in 1 mg/mL dsRNA solution (5.5 mM KH_2_PO_4_, 2.1 mM NaCl, 4.7 mM NH_4_Cl, 3 mM spermidine). Fluorescein 5-isothiocyanate (FITC) dye was used to confirm uptake. dsGFP served as a control. Following soaking, nematodes were washed and RNA extracted to assess silencing efficiency by RT-qPCR. Treated nematodes were either transferred to *B. cinerea* PDA plates or inoculated into seedlings stem; after 7 days, samples were collected for gene expression analysis as described. The treatment groups were designated as: dsBxyltlp5 (single-gene silencing), dsBxyltlp6 (single-gene silencing), and dsBxyltlp5&6 (dual-gene silencing). A dsRNA targeting the GFP gene was used as a control, named dsGFP group. Five biological replicates were conducted per group.

### Head-swinging behavior and chemotaxis assays

*B. cinerea* mycelia were suspended in M9 buffer (per liter: 3 g KH_2_PO_4_, 6 g Na_2_HPO_2_, 5 g NaCl, 1 mM MgSO_4_) and washed thoroughly. One hundred RNAi-treated nematodes were incubated in 100 µL fungal suspension at room temperature for 30 min in 96-well plate. Nematode behavior was observed under a stereomicroscope, and head-swing frequency was recorded using a manual counter. One head swing was defined as movement of the nematode head from the midline to one side and back to the midline. Counting began when the timer was started. The number of head swings within 1 min was recorded for each individual. Ten individuals were randomly selected for observation in each well. The assay was repeated with M9 buffer supplemented with 50 mM D-glucose, laminaripentaose, or fructose. Each treatment had twenty biological replicates.

Chemotaxis assays were performed on 0.5% agar plates (9 cm). Plates were divided into three zones: a central release point (~300 nematodes) and two opposing test zones (~3 cm from center). One zone was spotted with 10 µL of 50 mM test compound (D-glucose, laminaripentaose, or fructose) and the other with 10 µL M9 buffer (control). Plates were incubated at 25 °C in the dark for 1 h before counting nematodes within 1.5 cm of each spot under a stereomicroscope. Each treatment had five biological replicates.

### Fungal endophytic community analysis

To determine the role of BxylTLP6 in PWN pathogenicity, RNAi-treated nematodes were inoculated onto greenhouse-grown seedlings. Two treatments were included: seedlings inoculated with dsGFP-treated PWN (dsGFP group) and seedlings inoculated with dsBxylTLP6-treated PWN (dsBxylTLP6 group). Each treatment consisted of 15 seedlings. After inoculation, seedlings were monitored daily. A seedling was considered dead when all needles had wilted and turned yellow. The time to death for each seedling was recorded and used to generate survival curves.

To investigate whether BxylTLP6 influences the pine endophytic fungal community during nematode infection, we selected stem tissues collected 7 days post-PWN inoculation for ITS sequencing. This time point represents a stable stage of infection at which nematodes have completed host invasion and begun to proliferate within the stem, providing sufficient time for the effects of nematode-secreted TLPs to accumulate and potentially alter the resident fungal community. Four experimental treatments were implemented: seedlings inoculated with dsGFP-treated PWN (dsGFP group), seedlings inoculated with dsBxyltlp6-treated PWN (dsTLP group), seedlings injected with purified BxylTLP6 protein (BxylTLP6 group), control seedlings injected with heat-inactivated protein (HI-BxylTLP6 group). Inoculation procedures for nematodes were as above. For protein injection, a ~3 mm diameter hole was drilled 10–15 cm above the soil line at 45°, and a modified pipette tip (beveled end) containing 100 µL protein solution (10 mg/mL) was inserted and sealed with parafilm. Each treatment had five biological replicates, each consisting of five seedlings.

At 7 dpi, 2 cm stem segments (3 cm above inoculation) were collected and stored at −80 °C. The genomic DNA of all the samples was extracted using the CTAB/SDS method^41^, quality-checked by agarose gel electrophoresis and NanoDrop2000 (ThermoFisher). The ITS1 region of the fungal ITS gene were amplified with primer pairs in Supplementary Table 1 by T100 Thermal Cycler PCR thermocycler (BIO-RAD, USA). The PCR reaction mixture including 4 μL 5× Fast Pfu buffer, 2 μL 2.5 mM dNTPs, 0.8 μL each primer (5 μM), 0.4 μL Fast Pfu polymerase, 10 ng of template DNA, and ddH_2_O to a final volume of 20 µL. PCR amplification cycling conditions were as follows: initial denaturation at 95 °C for 3 min, followed by 27 cycles of denaturing at 95 °C for 30 s, annealing at 55 °C for 30 s and extension at 72 °C for 45 s, and single extension at 72 °C for 10 min, and end at 4 °C. The PCR product was extracted from 2% agarose gel and purified using the PCR Clean-Up Kit (YuHua, Shanghai, China) according to manufacturer’s instructions and quantified using Qubit 4.0 (ThermoFisher). Purified amplicons were pooled in equimolar amounts and paired-end sequenced on an Illumina Nextseq2000 platform (Illumina, San Diego, USA) according to the standard protocols by Majorbio Bio-Pharm Technology Co. Ltd. (Shanghai, China).

Raw FASTQ files were de-multiplexed using an in-house perl script, and then quality-filtered by fastp version 0.19.6^42^ and merged by FLASH version 1.2.7^83^ with the following criteria: the reads were truncated at any site receiving an average quality score of <20 over a 50 bp sliding window, and the truncated reads shorter than 50 bp were discarded, reads containing ambiguous characters were also discarded; only overlapping sequences longer than 10 bp were assembled according to their overlapped sequence. The maximum mismatch ratio of overlap region is 0.2. Reads that could not be assembled were discarded; Samples were distinguished according to the barcode and primers, and the sequence direction was adjusted, exact barcode matching, 2 nucleotide mismatches in primer matching. Then the optimized sequences were clustered into operational taxonomic units (OTUs) using UPARSE 7.1^84^ with 97% sequence similarity level. The most abundant sequence for each OTU was selected as a representative sequence. The OTU table was manually filtered, i.e., chloroplast sequences in all samples were removed. To minimize the effects of sequencing depth on alpha and beta diversity measure, the number of ITS gene sequences from each sample were rarefied to 43,867. The taxonomy of each OTU representative sequence was analyzed by RDP Classifier version 2.2 against the ITS gene database (eg. Unite) using confidence threshold of 0.7. The taxonomic information for the OTUs is provided in Supplementary Data 4.

Bioinformatic analysis was carried out using the Majorbio Cloud platform (https://cloud.majorbio.com)^85^. Based on the OTU information, rarefaction curves and alpha diversity indices including observed OTUs, Chao1 richness, Shannon index and Good’s coverage were calculated with Mothur v1.30.1^86^. Beta diversity was analyzed by NMDS (Bray-Curtis), with group differences tested by ANOSIM. Heatmaps (top 50 genera) were generated using average linkage clustering after log_10_ transformation. Differential OTUs were identified by Student’s *t* test with FDR correction. Co-occurrence networks (top 100 OTUs) were constructed using Spearman correlation (|r|≥ 0.5, *p* < 0.05).

Functional guilds were predicted with FUNGuild. To aid interpretation of FUNGuild assignments, it should be noted that several fungal taxa possess multiple ecological lifestyles. As a result, FUNGuild assigns composite guilds (e.g., “Plant pathogen-Wood saprotroph”) when taxa in the same genus or family have been described as expressing more than one trophic mode. This explains why “saprotroph” appears across multiple categories. Because our system does not involve mammals, all annotations related to animal pathogens were removed. Only functional guilds whose total relative abundance exceeded 1% were retained for comparative analyses among treatments.

### Statistical analysis

Comparisons between two groups were conducted using an unpaired independent-samples *t*-test, whereas comparisons among more than two groups were carried out using one-way ANOVA followed by Tukey’s post hoc test. Survival curves were generated using the Kaplan–Meier method, and differences between two groups were assessed with the log-rank test. A *P* value of <0.05 was considered statistically significant (*P < 0.05, **P < 0.01). Statistical analyses were performed using SPSS version 19.0. Graphs were created in GraphPad Prism version 9.5.1, and final figures were assembled in Adobe Illustrator version 26.0 and Adobe Photoshop version 23.2.

## Results

### Analysis of the TLP gene family in nematodes

We conducted a genome-wide survey of 160 nematode genomes and identified 203 *tlp* open reading frames (ORFs) from 58 species (Supplementary Data 2). *Tlp* genes were common in free-living nematodes (including those isolated from soil and decaying plant tissues), with 38 of 56 species (68%) collectively encoding 147 genes. In insect-parasitic nematodes, 2 of 7 species (29%) contained 6 *tlp* genes, whereas 8 of 12 insect-associated species (67%) possessed 33 genes. Plant-parasitic nematodes showed an intermediate pattern, with 9 of 20 species (45%) harboring a total of 16 *tlp* genes. Among 65 mammal-associated nematodes, only *Caenorhabditis bovis*, a free-living species isolated from Zebu cattle ears, harbored a single *tlp* gene; no *tlp* genes were detected in any other mammal-associated species.

**Fig. 1:**
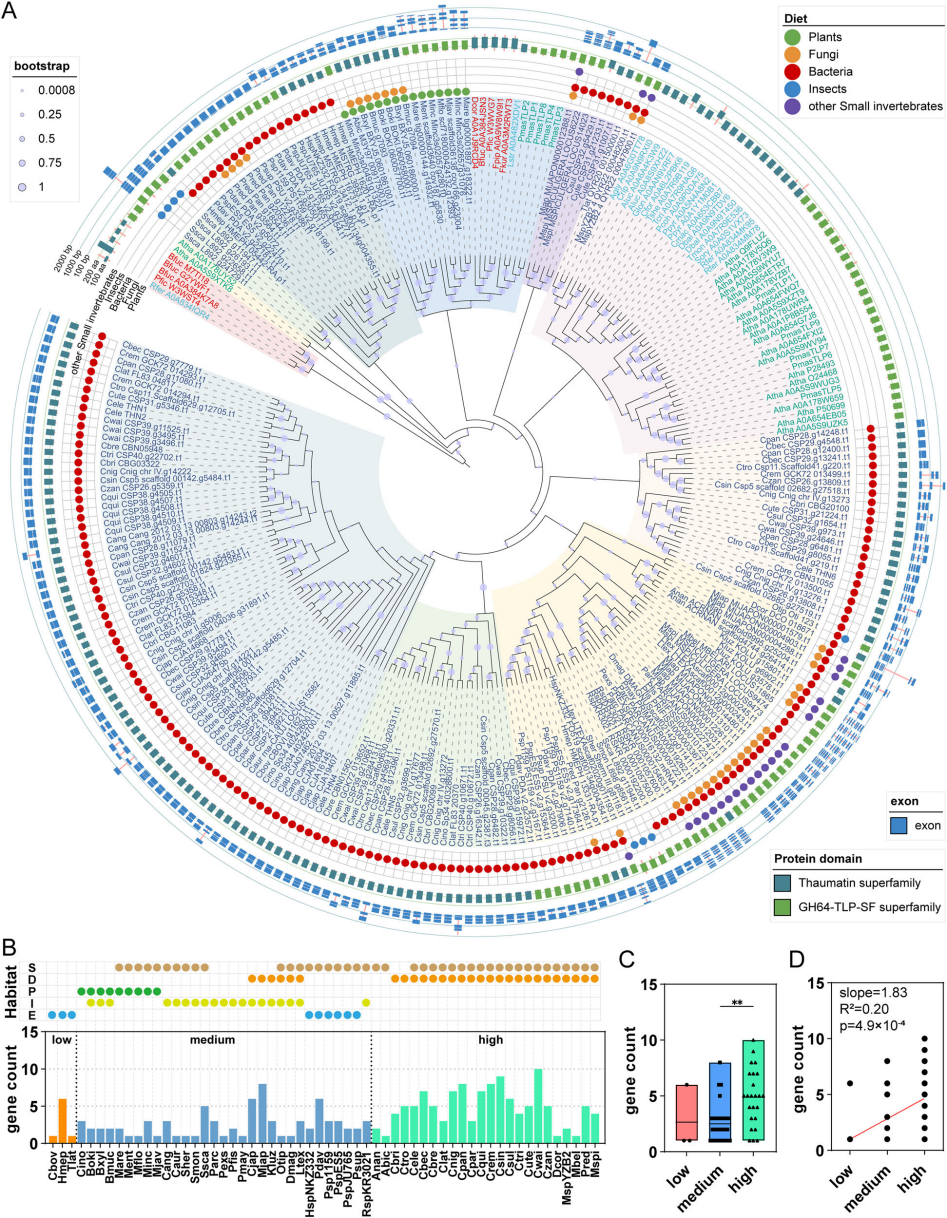
Analysis of the TLP gene family in nematodes. **A** Phylogenetic tree of TLPs protein. The tree was rooted using the midpoint method. Eleven distinct branch clusters were identified with different light-colored arcs, and bootstrap values displayed as light purple dots on branches reflect node support. Adjacent to the tree, colored dots indicated the ecological diet linked to each TLP: the green color represents plants, the orange color represents fungi, the red color represents bacteria, the blue represents insects, and the purple color represents other small invertebrates. The colored blocks denote conserved protein domains: the dark teal color represents Thaumatin superfamily and the green color represents GH64-TLP-SF superfamily. The outermost blue blocks represent exon regions, with the length of each block corresponding to the coding sequence length of exons. **B** The count of *tlp* genes in nematodes across habitats with different microbial-diversity classes. Light brown, orange, green, yellow, and blue dots were used to mark soil (S), decaying plant material (D), plants (P), insects (I), and E (extreme environments), respectively. **C** The differential analysis of the count of TLP genes in nematodes across different microbial-diversity classes. **D** Correlation analysis of the number of TLP genes in nematodes across different microbial-diversity classes based on a simple linear regression.

We constructed a phylogenetic tree using TLP amino acid sequences from plants, insects, and fungi together with the TLPs identified from nematodes (Fig. 1A). In addition, nematode sequences were annotated according to their dietary categories. All TLPs from plant-parasitic nematodes grouped within Cluster 5, together with TLPs from five plant-pathogenic fungi, one herbivorous insect, and five *P. massoniana* sequences. The presence of taxonomically diverse TLPs in the same well-supported clade suggests a complex evolutionary history. Substantial variation in exon-intron structures, ranging from fully intron-less genes to those with up to seven introns, reflects lineage-specific evolutionary histories. Intron gain and loss are well-documented to influence gene expression features, including transcriptional efficiency, alternative splicing potential, and mRNA stability^87–89^, suggesting that these structural differences may underlie differences in regulatory capacity among TLP genes (Fig. 1A).

We observed a significant positive association between habitat microbial diversity and TLP gene family size. We grouped the nematodes according to habitat type and quantified the number of TLP genes present in each species (Fig. 1B). When microbial-diversity class (low, medium, high) was encoded as an ordinal variable, Spearman’s rank correlation (ρ = 0.46, *p* = 2.9 × 10^−4^) indicated that species inhabiting more microbially diverse environments tend to possess more TLP genes. Consistently, a Kruskal–Wallis test revealed significant differences in TLP copy number among the three diversity classes (H = 12.54, *p* = 0.0019). Post hoc pairwise comparisons showed that species from high-diversity habitats harbor significantly more TLP genes than those from medium-diversity habitats (*p* = 0.0018), whereas differences involving the low-diversity class were not significant, likely due to the small number of species in this category (*n* = 3) (Fig. 1C). A simple linear regression further supported a monotonic increase in TLP copy number along the microbial-diversity gradient (slope = 1.83, R^2^ = 0.20, *p* = 4.9 × 10^−4^), corresponding to an average gain of almost two TLP genes for each step from low to medium and from medium to high microbial diversity (Fig. 1D). Together, these analyses suggest that species occurring in more microbially diverse habitats tend to harbor larger TLP gene families. This pattern further implies that TLPs may play an important role in mediating interactions between nematodes and the microbial communities they encounter.

### Identification of anti-fungal activity in secreted TLP protein from PWN

**Fig. 2:**
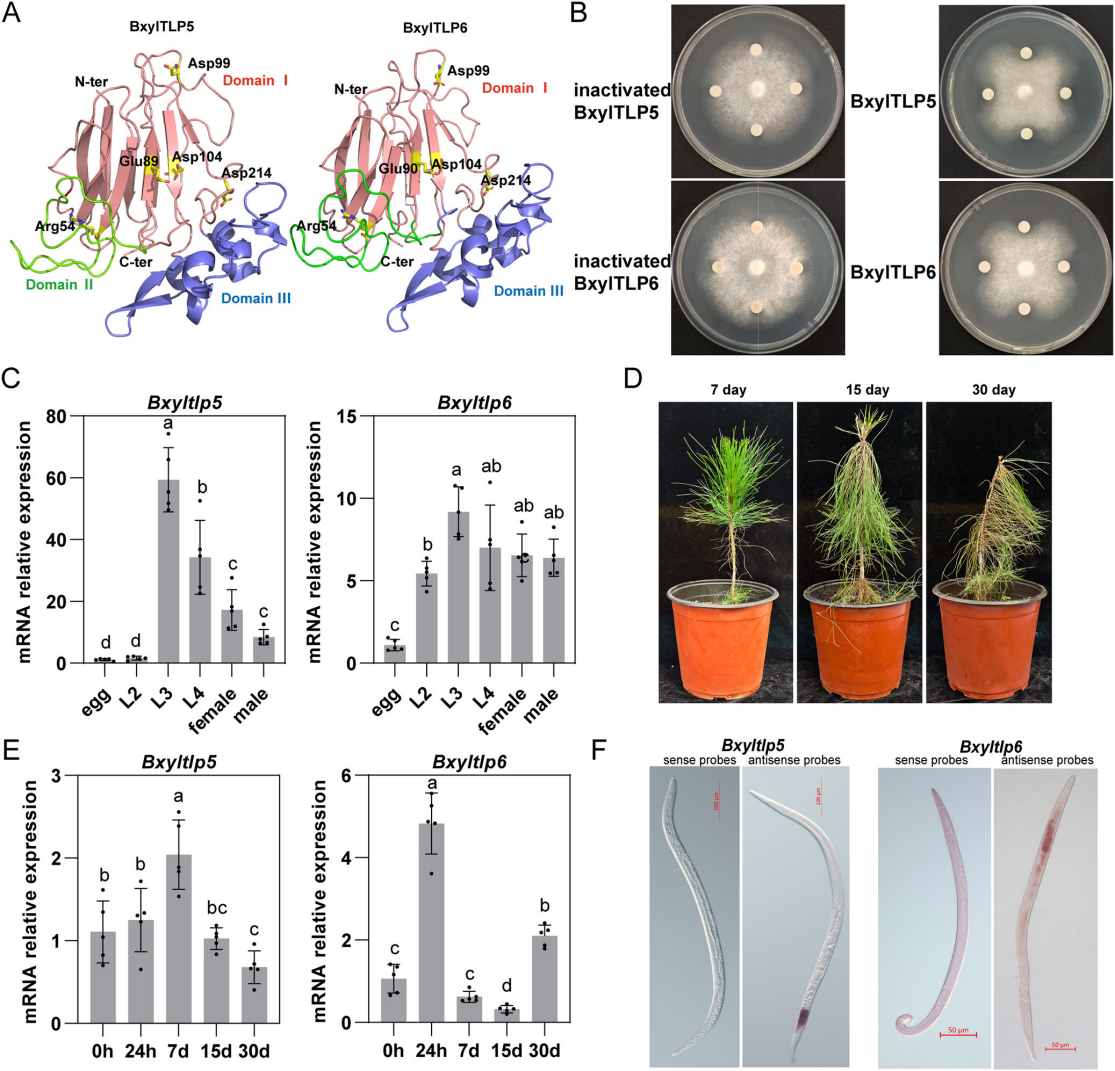
Identification of anti-fungal activity in secreted TLP protein from PWN. **A** Predicted three-dimensional structures of BxylTLP5 and BxylTLP6 generated by AlphaFold. The canonical TLP domain corresponds to Domain I, shown in red, which contains the conserved β-sandwich fold characteristic of TLP family proteins. Domain II (green) and Domain III (blue) represent additional structural regions extending from the core thaumatin-like domain. Conserved functional residues potentially involved in β-1,3-glucanase activity are highlighted in yellow. **B** Antifungal activities of recombinant BxylTLP5 and BxylTLP6 proteins against *B. cinerea*. **C** mRNA expression levels of *Bxyltlp5* and *Bxyltlp6* at different developmental stages of the PWN. **D** Dynamic disease phenotypes of two-year-old *P. massoniana* seedlings in the greenhouse following inoculation with PWN. **E** Temporal expression patterns of Bxyltlp5 and Bxyltlp6 genes at different infection stages after nematode inoculation of *P. massoniana* seedlings. **F** In situ hybridization in L4 larvae and adult nematodes cultured on *B. cinerea* showing localization of *Bxyltlp5* and *Bxyltlp6* mRNAs. The data are presented as means ± SD. Comparisons were carried out using one-way ANOVA followed by Tukey’s test. Different letters indicate significant differences.

The two TLP genes identified in PWN were designated as Bxyltlp5 (*BXYJ5.060186000*) and Bxyltlp6 (*BXYJ5.060186100*). The two TLPs share 59.85% amino acid identity, a level of similarity that is lower than typically observed for recently duplicated paralogs. Combined with the phylogenetic placement of these sequences, where they do not form a species-specific clade but instead cluster with TLPs from diverse taxa, this pattern indicates that the two genes are more likely to represent ancient orthologs retained from deeper evolutionary lineages rather than the result of recent, species-specific gene duplication. Multiple sequence alignment and secondary structure prediction revealed that both proteins contain 14 conserved cysteine residues predicted to form seven disulfide bonds, contributing to protein stability (Supplementary Fig. 1). Structural modeling using AlphaFold demonstrated that both BxylTLP5 and BxylTLP6 share the typical architecture of thaumatin-like protein family. A cleft located between Domain I and Domain II harbors conserved functional residues, Arg54, Glu89/90, Asp104, and Asp214, which are predicted to form a putative catalytic site associated with β-1,3-glucanase activity (Fig. 2A). Recombinant BxylTLP5 and BxylTLP6 proteins were successfully expressed and purified using a prokaryotic expression system (Supplementary Fig. 2). Enzymatic activity assays revealed β-1,3-glucanase activity levels of 0.224 ± 0.004 U/mg for BxylTLP5 and 0.168 ± 0.002 U/mg for BxylTLP6, respectively (Supplementary Fig. 3). Antifungal sensitivity assays using *B. cinerea* demonstrated clear inhibition zones around filter paper discs soaked with the recombinant proteins, confirming their antifungal activity (Fig. 2B). These findings indicate that both BxylTLP5 and BxylTLP6 display β-1,3-glucanase activity toward fungal cell walls and exhibit direct antifungal activity.

Then, we quantified their expression during fungal feeding on *B. cinerea* (Fig. 2C), revealing stage-specific patterns: *Bxytlp5* peaked in L3 larvae with progressive downregulation toward adulthood, while *Bxyltlp6* maintained stable expression across larval-adult stages, with both genes minimally transcribed in eggs. Subsequently, we inoculated the nematodes isolated from *B. cinerea* into *P. massoniana* seedlings to observe changes in *tlps* gene expression (Fig. 2D, E). For *Bxyltlp5*, expression peaked at 7 days post-inoculation, significantly exceeding the levels observed during the *B. cinerea*-feeding phase, but dropped below this baseline by day 30. In contrast, *Bxyltlp6* showed significantly higher expression on days 1 and 30 relative to the fungal-feeding phase, whereas its expression on day 15 was significantly lower. Notably, *Bxyltlp6* exhibited larger overall fold-change amplitudes than *Bxyltlp5* across these time points.

In situ hybridization was performed on L4 larvae and adult nematodes during fungal feeding on *B. cinerea*. It localized *Bxyltlp5* specifically to the posterior intestinal epithelia and *Bxyltlp6* to the anterior intestinal region and esophageal glands (Fig. 2F). These tissues represent major secretory sites in PWNs, and proteins produced in these regions can be released into the external environment through the stylet or via intestinal secretions^90,91^. Collectively, following the invasion of pine seedlings by PWN, both TLP genes undergo dynamic expression reprogramming in response to shifting host physiological states, with differential expression patterns observed between *Bxytlp5* and *Bxyltlp6*.

### Function of BxylTLPs in the fungal foraging process of PWN

It is generally accepted that PWN primarily feeds on host plant cells during the early stages of infection in healthy pine trees. As the host begins to weaken or eventually dies, the nematode gradually shifts to feeding on fungi^35–37^. However, direct evidence for a shift in the nematode’s feeding preference under these changing conditions has been lacking.

**Fig. 3:**
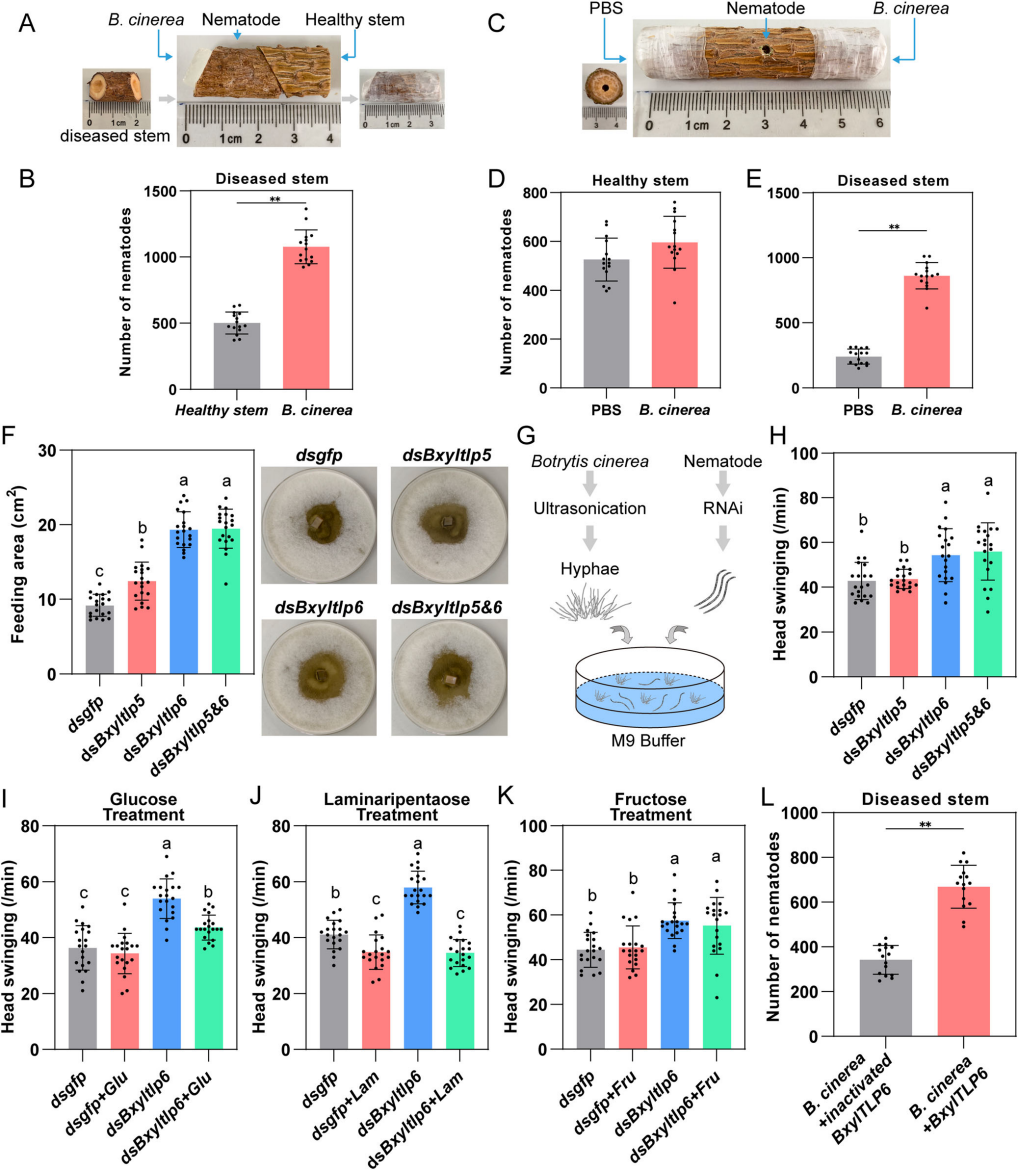
Function of BxylTLPs in the fungal-foraging process of the PWN. **A** Schematic diagram of behavioral choice assays of PWNs between healthy stem segments and B. cinerea. **B** Differences in the number of PWNs aggregated in healthy stem segments versus *B. cinerea*. **C** Behavioral choice assays of PWNs between control and *B. cinerea* mycelia in healthy and dead stem segments. **D** Comparison of PWN aggregation at opposite ends of healthy stem segments containing either control (PBS) or *B. cinerea* mycelia. **E** Comparison of PWN aggregation at opposite ends of dead stem segments containing either control (PBS) or *B. cinerea* mycelia. **F** Feeding areas of PWNs on *B. cinerea* plates following RNAi-mediated knockdown of *tlp* genes. dsgfp, control; dsBxyltlp5 and dsBxyltlp6, knockdown of *Bxyltlp5* and *Bxyltlp6* respectively; dsBxyltlp5&6, simultaneous knockdown of both genes. **G** Schematic diagram of the foraging behavior assay. **H** Effect of *tlp* genes silencing on the head-swinging frequency of PWNs. **I**–**K** Effects of adding glucose, fructose, or laminaripentaose on PWN head-swinging frequency. **L** Aggregation of PWNs in dead wood segments between the control side *(B. cinerea* mycelia supplemented with heat-inactivated recombinant BxylTLP6 protein) and the treatment side (*B. cinerea* mycelia supplemented with recombinant BxylTLP6 proteins). The data are presented as means ± SD. Comparisons in (**B**), (**D**), (**E**) and (**L**) were conducted using independent-samples *t*-test. *P < 0.05, **P < 0.01. Comparisons in (**F**), (**H**–**K**) were carried out using one-way ANOVA followed by Tukey’s test. Different letters indicate significant differences.

To address this gap, we conducted a series of behavioral assays to evaluate the nematode’s food source preference. We first positioned *B. cinerea* mycelia and healthy *P. massoniana* seedling stem segments at opposite ends of a wilted pine segment, placing nematodes in the center (Fig. 3A). The nematodes exhibited a strong preference for the fungal side over the fresh pine tissue (Fig. 3B). Then, we prepared stem segments from fresh, healthy seedling and drilled small holes at both ends. One end was filled with PBS as control, and the other with a suspension of *B. cinerea* mycelia. Nematodes were introduced to the center of the stem (Fig. 3C). No significant difference was observed in nematode distribution between the two ends (Fig. 3D). Then, we repeated the experiment using stem segments from wilted seedlings previously infected with PWN. In contrast to the previous result, significantly more nematodes migrated toward the side containing *B. cinerea* mycelia (Fig. 3E). These results suggest that PWN prefers feeding on fungi, but it does not actively seek fungal resources within healthy pine hosts. Only when host condition declines do the nematode shift its preference toward fungal consumption.

To functionally characterize BxylTLP5 and BxylTLP6 during the fungal-feeding stage of PWN, we conducted RNAi-mediated knockdown experiments. Following 36-h soaking of L3-stage larvae, intense FITC fluorescence was observed in the intestinal lumen of most nematodes (Supplementary Fig. 4A), confirming effective environmental uptake via aqueous immersion. Quantitative PCR revealed a significant reduction in the relative mRNA levels of both *Bxyltlp* genes compared to the control group (Supplementary Fig. 4B). Notably, silencing one gene alone did not affect the expression of the other. The RNAi-treated nematodes were then transferred to *B. cinerea* culture plates, where the silencing effect of the *Bxyltlp* genes persisted for more than 7 days (Supplementary Fig. 4C).

We found that nematodes treated with *Bxytlp* gene-targeting dsRNA exhibited significantly larger feeding areas on *B. cinerea* plates compared to the control group (Fig. 3F). Notably, the dsBxyltlp6 and dsBxyltlp5&6 groups displayed larger feeding areas than the dsBxyltlp5 group, suggesting that BxylTLP6 may play a more prominent role in modulating feeding behavior. However, there were no significant differences in nematode body length or population size among the different groups (Supplementary Fig. 5), indicating that gene silencing affected feeding behavior without compromising overall nutrient uptake. To determine feeding-associated behavioral changes, PWN subjected to RNAi treatments were introduced into M9 buffer containing *B. cinerea* hyphae, with locomotion patterns quantified (Fig. 3G). The dsBxyltlp6 and dsBxyltlp5&6 groups exhibited significantly elevated head swinging frequencies compared to controls and dsBxyltlp5-treated nematodes (Fig. 3H). As head swinging represents a fundamental foraging behavior mediating both environmental cue perception and locomotor activity, its amplification typically indicates intensified food-seeking motivation. These results suggest that RNAi-mediated silencing of Bxyltlp6 appears to impair chemosensory perception of food signals while simultaneously enhancing exploratory locomotion.

Given the capacity of nematode-secreted TLPs to degrade fungal cell walls, we hypothesized that *Bxyltlp6* silencing would reduce fungal-derived food signals. As TLPs primarily generate oligosaccharides and glucose monomers through cell wall degradation^48,49^, we supplemented dsGFP control and dsBxyltlp6 groups with exogenous glucose and quantified head swinging frequencies. Glucose supplementation did not alter head swinging in the dsGFP group. In contrast, dsBxyltlp6 nematodes exhibited significantly reduced head swinging following glucose treatment compared to glucose-untreated dsBxyltlp6 counterparts, though frequencies remained higher than dsGFP controls (Fig. 3I). Evolutionary analysis indicates nematode TLPs share homology with GH64 β-1,3-glucanases, which predominantly release laminaripentaose as a catalytic product (Supplementary Fig. 6A). We therefore tested laminaripentaose supplementation. Both control and dsBxyltlp6 groups showed significantly suppressed head swinging upon laminaripentaose addition (Fig. 3G). To determine whether supplemented saccharides functioned as chemosensory signals or nutritional substrates in this experimental system, parallel experiments employed fructose, a nematode nutrient absent from fungal wall degradation. Fructose supplementation, by contrast, had no effect in either the dsGFP or dsBxyltlp6 groups. Head-swinging frequencies in dsBxyltlp6 nematodes were not reduced by fructose addition and remained significantly different from dsGFP controls (Fig. 3K). Concurrently, chemotaxis assays confirmed chemoattraction of PWN to glucose, laminaripentaose, and fructose (Supplementary Fig. 6B, C).

We also repeated the above behavioral preference assay of PWN within wood segments. We applied *B. cinerea* mycelial suspension to both ends of the wilted wood segment, with one end additionally supplemented with the recombinant BxylTLP6 protein and the other end supplemented with heat-inactivated protein. The results showed that the nematodes exhibited a stronger preference for the end treated with BxylTLP6 (Fig. 3L). This suggests glucose and laminaripentaose likely function as food-derived signaling molecules that elicit nematode taxis. Crucially, TLP silencing diminishes fungal cell wall-derived glucose and laminaripentaose, triggering intensified food-seeking behavior.

### Effects of BxylTLP on the endophytic fungal community in pine

**Fig. 4:**
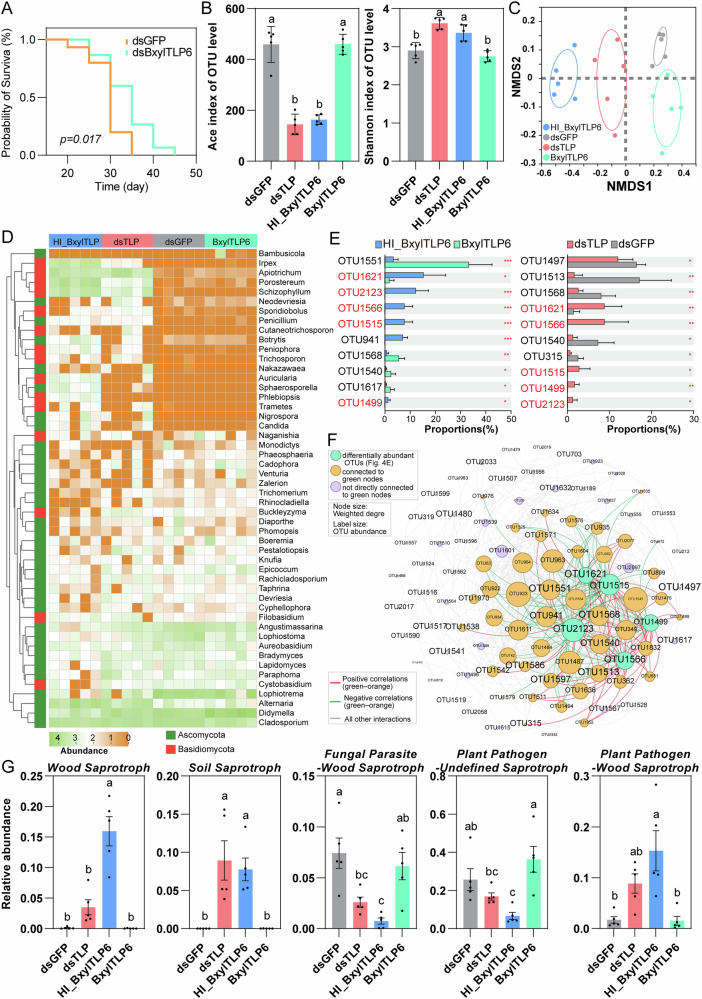
Role of BxylTLP6 in shaping the pine endophytic fungal community. **A** Survival curves of two-year-old *P. massoniana* seedlings in the greenhouse after inoculation PWNs treated by soaking in dsRNA targeting *gfp* (dsGFP, control) or dsRNA targeting *Bxyltlp6* (dsBxyltlp6). Differences between survival curves were evaluated using the Log-rank (Mantel–Cox) test. *P* = 0.017 indicates a statistically significant difference between groups. **B** ACE and Shannon indices of endophytic fungal communities in stem segments collected 5 cm above the inoculation site, 7 days post-inoculation. **C** Non-metric multidimensional scaling (NMDS) analysis of fungal communities based on Bray–Curtis dissimilarities (*n* = 5). **D** Heatmap of the top 50 most abundant genera. **E** Comparison of differentially abundant OTUs between groups. **F** Co-occurrence network of the top 100 most abundant OTUs. Green nodes represent the differentially abundant OTUs identified in Fig. 4E. Orange nodes represent OTUs that share at least one edge with the green nodes, whereas purple nodes correspond to OTUs without direct edges to any green node. Node size indicates the weighted degree of each OTU, and label size reflects OTU abundance. Red edges denote positive correlations between green and orange nodes, green edges denote negative correlations between these nodes, and gray edges represent interactions among all remaining OTUs. **E** Orange nodes represent OTUs correlated with these differentially abundant OTUs. Node color denotes the number of edges, and label font size indicates relative abundance. **G** Effects of BxylTLP6 on predicted fungal community functions. Functional guilds were assigned using FUNGuild. Because some fungal taxa exhibit multiple ecological lifestyles, FUNGuild may classify them into composite guilds (e.g., “Plant pathogen-Wood saprotroph”). Only guilds with a total relative abundance >1% were retained for comparison. The data are presented as means ± SD. Comparisons were carried out using one-way ANOVA followed by Tukey’s test. Different letters indicate significant differences.

To elucidate the functions of Bxyltlps during plant cell feeding, we inoculated *P. massoniana* seedling stems with PWNs subjected to RNAi treatments. Quantitative PCR confirmed sustained *tlp* gene silencing seven days after inoculation (Supplementary Fig. 7). In contrast to the observations in *B. cinerea* plate-feeding assays, *Bxyltlp* silencing did not significantly affect nematode migration distance, survival rates, or body length in pine hosts (Supplementary Fig. 8). Among the two TLP genes identified in *B. xylophilus*, Bxyltlp6 exhibited a much stronger transcriptional response during pine infection, and we therefore focused our subsequent analyses on *Bxyltlp6*. Disease progression monitoring revealed statistically equivalent mortality rates across all treatments; however, dsBxyltlp6-inoculated pines exhibited significantly delayed symptom development (Fig. 4A), indicating that although host mortality was unchanged, *Bxyltlp6* contributes to nematode-mediated disease development.

Given the antifungal properties of TLPs, we next examined whether they, like antimicrobial effectors of fungal pathogens, also influence the host mycobiome. To test this, greenhouse pine seedlings were injected with dsGFP-treated nematodes (dsGFP group), dsBxyltlp6-treated nematodes (dsTLP group), recombinant BxylTLP6 protein (BxylTLP6 group), or heat-inactivated BxylTLP6 protein (HI-BxylTLP6 group). Samples were collected after 7 days for ITS sequencing of fungal community diversity. We conducted ITS sequencing across all treatment groups. We obtained a total of 877,340 reads from 20 samples of seedling stems. These sequences were assigned to 1445 fungal operational taxonomic units (OTUs) (Supplementary Data 4). Analysis of fungal α-diversity revealed that fungal richness (Ace index) was significantly higher in the dsGFP and BxylTLP6 group compared to the HI- BxylTLP6 and dsTLP groups, with no significant difference observed between dsGFP and BxylTLP6, or between HI-BxylTLP6 and dsTLP (Fig. 4B). In contrast, fungal diversity (Shannon index) was significantly reduced in the dsGFP and BxylTLP6 groups relative to the HI-BxylTLP6 and dsTLP groups, with no significant differences between groups within each comparison (Fig. 4B). NMDS analysis showed that there was a clear separation among the four treatment groups (stress: 0.063, R = 0.87167, *P* = 0.001), indicating that different treatment groups had a significant impact on the composition of the fungal community (Fig. 4C). The primary differences in microbial community structure were distributed along the NMDS1 axis, showing a gradual shift from the HI-BxylTLP6 group, through the dsTLP group, to the dsGFP and BxylTLP6 groups. Moreover, along the NMDS1 axis, the BxylTLP6 group was more closely clustered with the dsGFP group. In addition, OTU-based Venn diagram analysis showed that 42 OTUs were shared exclusively between the HI-BxylTLP6 and dsTLP groups but absent in the BxylTLP and dsGFP groups, while 282 OTUs were uniquely shared between the BxylTLP and dsGFP groups and absent from the other two groups (Supplementary Fig. 9). This pattern suggests that the BxylTLP6 protein significantly altered the overall microbial composition.

We further examined fungal community structure across treatments. Phylum-level community profiling revealed that the pine endophytic mycobiome was dominated by *Ascomycota* and *Basidiomycota* across all treatments (Supplementary Fig. 10). Seedlings in the dsGFP and BxylTLP6 groups showed a strongly Ascomycota-dominated community with minimal Basidiomycota. In contrast, the dsTLP and HI-BxylTLP6 groups exhibited reduced Ascomycota and a marked increase in Basidiomycota (Supplementary Fig. 11). These patterns indicate that active BxylTLP6 suppresses *Basidiomycota* taxa within the host while maintaining or promoting the dominance of *Ascomycota*, thereby shaping the phylum-level structure of the pine endophytic mycobiome.

We analyzed the heatmap and cluster tree depicting the genus-level fungal community composition (Fig. 4D). Within the dsGFP and BxylTLP6 groups, genera *Irpex*, *Apiotrichum*, *Porostereum* and *Schizophyllum* exhibited significantly reduced relative abundances, suggesting potential direct suppression by the BxylTLP6 protein. Conversely, genera such as *Cadophora* and *Venturia* showed significantly increased relative abundances in these same groups, indicating potential indirect effects mediated by BxylTLP6. Further analysis of differentially abundant top 10 OTUs between groups demonstrated five OTUs with significantly decreased relative abundance in the HI-BxylTLP6 vs. BxylTLP comparison were also decreased in the dsGFP vs. dsTLP comparison. These overlapping OTUs included OTU1621 (*Alternaria* sp.), OTU2123 (*Porostereum spadiceum*), OTU1566 (*Apiotrichum montevideense*), OTU1515 (*Schizophyllum radiatum*), and OTU1499 (order Saccharomycetales) (Fig. 4E). Co-occurrence analysis of the top 100 abundant OTUs revealed that the BxylTLP groups contained more edges, indicating that injection of the BxylTLP6 protein increased the complexity of interactions among species within the community (Supplementary Fig. 12). Co-occurrence network analysis indicated that the five overlapping OTUs identified above, OTU1621, OTU2123, OTU1566, OTU1515, and OTU1499, possessed high relative abundance, high degree centrality values, and exhibited significant positive or negative correlations with most OTUs in the co-occurrence network (Fig. 4F). Collectively, these results suggest that BxylTLP6 may contribute to shifts in the fungal community by influencing key taxa and modulating overall community interactions.

To assess the functional implications of TLP-mediated changes in fungal communities, we annotated fungal OTUs using the FUNGuild database. Functional guild analysis revealed that the HI-BxylTLP6 and dsTLP groups exhibited similar functional profiles, while the dsGFP and BxylTLP6 groups also exhibited similar functional profiles (Supplementary Fig. 13). When comparing the dsTLP and dsGFP groups, only two guilds exhibited significant differences: the relative abundance of Soil Saprotrophs was substantially lower in the dsGFP group, whereas the Fungal Parasite-Wood Saprotroph guild was significantly enriched (Fig. 4G). The remaining three guilds showed no significant differences between these treatments. In contrast, a clear shift was observed when comparing HI-BxylTLP6 and BxylTLP6 treatments, where all five functional guilds differed significantly (Fig. 4G). The Fungal Parasite-Wood Saprotroph and Plant Pathogen-Undefined Saprotroph guilds were more abundant in the presence of active BxylTLP6, whereas the Wood Saprotroph, Soil Saprotroph, and Plant Pathogen-Wood Saprotroph guilds were markedly reduced. These patterns highlight that the activity of BxylTLP6 is associated with a pronounced restructuring of fungal functional groups. Notably, no significant differences were detected between the dsTLP and BxylTLP6 groups in Wood Saprotroph, Fungal Parasite-Wood Saprotroph and Plant Pathogen-Wood Saprotroph guilds. This may reflect incomplete RNAi silencing, resulting in residual BxylTLP6 activity in dsTLP nematodes, and further suggests that nematode-mediated modulation of fungal functional guilds is modulated not only by BxylTLP6 but also by additional effectors and host-mediated indirect responses. Collectively, these findings indicate that active BxylTLP6 is associated with a shift toward reduced saprotrophic guilds and increased representation of guilds with parasitic or pathogenic lifestyles, although its effects likely act in concert with broader nematode- and host-derived factors that together modulate the functional architecture of the pine endophytic mycobiome.

We further examined the effects of BxylTLP6 on fungi with different ecological functions isolated from pine tissues, which included five putative blue-stain fungi strains (*Ophiostoma ips*), which commonly co-occur with pine wilt disease and can serve as food sources for PWN; two putative plant-pathogenic fungi strains (*Botryosphaeria dothidea*), which may interact antagonistically with the nematode; and three putative wood-decaying fungi strains (*Irpex lacteus* and *Phanerochaete concrescens*), which degrade lignocellulose. Antifungal assays showed that BxylTLP6 exhibited varying degrees of inhibitory activity against all tested fungi except *I. lacteus* FXY528 (wood-decaying fungi) and *B. dothidea* FXY573 (plant pathogen fungi) (Supplementary Fig. 14). This broad-spectrum antifungal activity is consistent with previous reports on TLPs protein^61^.

We also assessed the feeding capacity of PWN on these fungi (Supplementary Fig. 15A, B). The nematodes were able to feed and reproduce on four *O. ips* strains (blue-stain fungi) and on *B. dothidea* FXY94 (plant-pathogenic fungus), although their reproductive rates were lower than when feeding on *B. cinerea*. On *O. ips* FXY778 (blue-stain fungus), *P. concrescens* FXY475 (wood-decaying fungus), and *I. lacteus* FXY618 (wood-decaying fungus), nematode populations increased during the first 10 days after inoculation but subsequently declined. Hyphal damage was evident on these plates, indicating that although nematodes could feed on these fungi, they were unable to complete their life cycle. In contrast, when inoculated onto *I. lacteus* FXY528 (wood-decaying fungus) and *B. dothidea* FXY573 (plant-pathogenic fungus), nematode numbers declined sharply and no feeding traces were observed under the microscope, suggesting that these fungi exert strong antagonistic effects on the nematode. Notably, these two isolates also exhibited resistance to the antifungal activity of BxylTLP6. Together, these results indicate that BxylTLP6 may contribute to the ability of PWN to exploit a subset of blue-stain and plant-pathogenic fungi as food resources, whereas wood-decaying fungi appear unsuitable for supporting nematode development under our experimental conditions.

## Discussion

In plants, TLP genes are widespread and exhibit high diversity, with 1816 plant TLPs identified across 187 species in the UniProt database alone, predominantly in angiosperms^61^. In contrast, fungi and insects harbor far fewer TLP genes, and TLP genes remain undetected in some insect lineages^61^. Our research indicates that the TLP gene family is not ubiquitous among nematodes. TLP ORFs containing the complete thaumatin domain were identified in only 58 out of 160 nematode genomes analyzed. The identified nematode TLPs are primarily distributed in free-living or parasitic nematodes originating from environments such as soil, plants, or insects. Previous studies suggest that this evolutionary pattern is modulated by ecological demands driving functional divergence of proteins^60,92^. A notable aspect is plant-pathogen co-evolution^93^, which has likely influenced TLP diversification. Recent studies have revealed that TLP genes may have integrated into the genomes of the greenhouse whitefly (*T. vaporariorum*) and the sweet potato whitefly (*B. tabaci*) via horizontal gene transfer (HGT)^69^. In *T. vaporariorum*, this gene enhances defense against fungal pathogen infection, while in *B. tabaci*, the TLP-encoded effector protein suppresses jasmonic acid (JA)-mediated defense responses in host plants, thereby facilitating host adaptation and evolution, reflecting a potential trend of functional diversification in insects. In our phylogenetic analysis, TLPs from PPNs clustered together with those from plant-pathogenic fungi, herbivorous insects, and plants. This pattern suggests a more complex evolutionary history, potentially involving an ancient shared ancestry, lineage-specific gene retention, horizontal gene transfer, or other evolutionary processes that can lead to phylogenetic intermixing across divergent taxa.

Furthermore, the number of TLP genes present in a nematode correlates with its habitat: nematodes inhabiting environments with higher microbial abundance tend to possess a greater number of TLP genes in their genomes. Previous studies have shown that the evolution of antimicrobial effectors maybe modulated by the microbial diversity of the environment^6^. In low-diversity endosphere niches, obligate biotrophs such as Albugo candida tend to evolve highly selective effectors that target a small number of antagonistic microbes^94^. In contrast, foliar pathogens like Zymoseptoria tritici and Ustilago maydis inhabit the microbially dense and competitive plant surface, where broad-spectrum antimicrobial effectors are advantageous^95^. Previous studies on nematode genomes have demonstrated that gene loss and gene family expansion in nematode genomes are closely linked to their lifestyles^96,97^. The G protein-coupled receptor (GPCR) family, as important cell surface receptors, has undergone contraction in plant-parasitic nematodes, whereas in the free-living nematode *C. elegans*, this family has expanded nearly tenfold, with the majority being olfactory receptors^98,99^. Studies on other gene families in *B. xylophilus* have also reported expansions in the repertoire of genes involved in detoxification processes, which may be associated with its complex lifestyle^100^. Based on this relationship between gene expansion and lifestyle, we propose that when the richness or diversity of microorganisms in the habitat is higher, nematodes tend to harbor a greater variety of TLP genes, which may enhance their adaptive capacity to the microbial ecological environment.

During PWN infection of pine trees, the nematode engages in various types of interactions with the resident fungal communities, one of the most crucial being that some of these fungi serve as food resources. Our study reveals that when both fungal food sources and healthy pine cells are accessible, PWN exhibits a preference for fungi. This preferential selection is likely attributed to the fact that feeding on plant cells entails greater energy expenditure, which is associated with the need to evade the defense responses of pine trees and undergo detoxification processes in the nematode^101,102^. However, when PWN is confined solely within healthy pine stems, it does not exhibit active searching for fungal food resources. We speculate that in this scenario, perceiving abundant healthy plant cells, the nematode avoids expending additional energy on migratory movement to locate fungi. Furthermore, studies on plant endophytes indicate that the abundance of endophytic microbes in aboveground plant parts is significantly lower than in soil and other saprophytic environments^103–105^. Therefore, actively seeking out scarce fungal resources does not appear to be the optimal strategy when the plant is healthy. A similar foraging pattern has been reported in *C. elegans*. *C. elegans* behavioral responses are often dominated by a single environmental variable, typically food density measured as bacterial units per surface area, while largely ignoring other factors like food type or biomass^96,106^. Rules of thumb are behavioral algorithms that approximate optimal behavior while lowering cognitive and sensory costs^106^. When choosing between different types of bacterial food, *C. elegans* also follows the principle of maximizing economic utility to select preferred food^106^.

The behavioral significance of PWN secreting TLP proteins to degrade fungal cell walls lies in its role in locating fungal food resources. Animals generally depend on signals released by food itself for resource localization. *C. elegans* can select among bacterial food sources based on olfactory and gustatory cues^96^. Root-parasitic nematodes can recognize root exudates to locate and parasitize host plants^107,108^. Our results show that glucose and oligosaccharides can attract PWN and regulate its foraging behavior. Studies on root-knot nematodes have shown that cell wall polysaccharides act as attractants^109,110^, and synthetic α-L-galactosyl-1,3-L-rhamnose also exhibits attractant properties for these nematodes^111^. For *C. elegans*, glucose and fructose initially function as repellents; however, the nematode can acquire chemotactic memory toward these sugars through associative learning^112^. Importantly, oligosaccharides are ubiquitous in the habitat of PWN. During feeding, PWN inserts its stylet into cells to ingest cellular contents while leaving the cell wall intact. Although fungal and plant cell walls differ in composition, both utilize polysaccharides as their structural backbones, with glucans being a key component^113^. Previous studies have reported that PWN secretes other plant cell wall-degrading enzymes, such as cellulases and pectate lyases^114,115^, which degrade plant cell walls into oligosaccharides^116,117^. The ability of PWN to recognize oligosaccharides contributes to our understanding of how it identifies regions with high densities of healthy pine cells and fungal cells. When the health status of the host pine declines or the tree dies, oligosaccharides generated by TLP-mediated degradation of fungi become the primary food signals for PWN. As observed in *C. elegans*, nematodes increase their movement speed during food search, slowing down upon reaching a food source. When food quality or density is high, they tend to dwell and feed locally; when food is depleted, they roam faster to expand their search area^96^. In this process, oligosaccharides generated by the degradation of fungal cell walls via TLPs secreted by the nematode serve as indicator signals of food resource density.

Our study provides evidence that PWN can modulate the structure of host fungal communities through the secretion of BxylTLP6. Both the purified protein treatment (BxylTLP6 group) and the nematode control (dsGFP group) exhibited similar patterns of community restructuring, characterized by decreased Shannon diversity but increased ACE richness. Because Shannon reflects both richness and evenness, whereas ACE is particularly sensitive to low-abundance OTUs, this divergence suggests an accumulation of low-abundance taxa accompanied by reduced evenness driven by increased dominance. The dsGFP and BxylTLP6 communities were strongly Ascomycota-dominated, with Basidiomycota contributing only a minor fraction, whereas Basidiomycota increased markedly in dsTLP and HI-BxylTLP6, a pattern consistent with higher evenness and, consequently, higher Shannon diversity. Basidiomycota are enriched in saprotrophic and ectomycorrhizal fungi, whereas Ascomycota encompass a broader range of guilds, including numerous plant pathogens, endophytes, saprotrophs and yeast-like fungi. At the genus level, declines in several Basidiomycota-associated wood-decay genera (e.g., *Irpex*, *Porostereum* and *Schizophyllum*), together with increases in other taxa (e.g., *Cadophora* and *Venturia*), suggest selective suppression coupled with secondary expansion of taxa that are tolerant of, or favored by, the altered niche. In addition, the 282 OTUs uniquely shared by dsGFP and BxylTLP6 but absent from dsTLP and HI-BxylTLP6 support the view that richness gains are largely driven by low-abundance taxa that persist or emerge following niche release. Although the ACE increase observed here differs from reports in *P. pinaster* and *P. massoniana*, where fungal richness typically declines after PWN infection^53,55^, the phylum-level directionality in our data, namely suppression of Basidiomycota alongside maintained or enhanced Ascomycota, accords with prior descriptions of PWN-associated mycobiome restructuring^53^.

BxylTLP6-mediated reshaping of pine endophytic fungal communities’ results in a notable increase in plant-parasitic and pathogenic fungi, alongside a marked decrease in wood-decaying fungi. The enrichment of pathogenic fungi such as *Saitozyma*, *Graphilbum*, *Diplodia*, and *Candida* is widely recognized as facilitating PWN infection by weakening host defenses during the nematode invasion stage^38,50,55,56^. At the same time, the increase of plant-parasitic and pathogenic fungi also provides fungal food resources for PWNs, particularly after the host pine begins to decline^38,50^. Therefore, we propose that the increase in plant-parasitic and pathogenic fungi may facilitate nematode infection while simultaneously enhancing the availability of fungal food resources during the late stages of infestation. The reduction of wood-decaying fungi is likely relevant to PWN survival during the late stage of infection, when host tissues weaken or die. Wood-decay fungi typically proliferate in decomposing wood^118,119^, yet PWNs generally do not feed on them. Among reported fungal food sources, only *P. subvermispora*, *F. roseus*, *Tyromyces* spp., and *P. ferruginosa* are potential decay fungi^38,49,120^. Moreover, wood-decay fungi can inhibit PWN survival^47,48^, and in our assays, two of the three tested species did not support completion of the nematode life cycle, while the third strongly suppressed PWN growth. These observations suggest that suppressing wood-decaying fungi reduces ecological stress for PWNs.

Crucially, we propose that limiting wood-decay fungi helps maintain the stability of the PWN niche as the host deteriorates. Reduced decay activity may preserve microhabitat integrity and allow other fungal guilds to occupy available niches. After host death, PWNs must survive within the decaying stem until they transfer to larvae or newly emerged adults of *Monochamus* beetles, their only natural vectors. When the beetles emerge and feed on healthy pine trees, the nematodes are transported to new hosts, enabling their spread, which is an essential step in the nematode’s life cycle^36^. Excessive wood decay, through lignocellulose degradation and alteration of wood chemistry, could jeopardize this transitional habitat^94^. Similar ecological dynamics have been reported in insect-fungus systems. For instance, early colonization by *Hylurgops palliatus* and *Monochamus sutor* influences the activity of wood-decaying fungi and affects later-arriving species^121^. Ambrosia beetles introduce fungal communities that suppress wood decay by outcompeting decay fungi^122^. And bark beetle-associated fungi similarly inhibit sapwood decomposition through microbial exclusion^123^. Termites provide further evidence: antifungal compounds in their saliva and gut (e.g., β-1,3-glucanases) inhibit the growth of certain wood-decaying fungi, thus highlighting a competitive dynamic between the insect and these microbes^124^. This concept is also supported by studies of fungal effectors, such as *V. dahliae* effector VdAMP3, which facilitates microsclerotia formation in decaying host tissues and functions to protect the fungus from opportunistic competitors^8^. By analogy, we propose that PWN may secrete antimicrobial effectors like TLPs to protect their niche from fungal competitors that emerge as host tissues decay and immunity wanes.

TLP-mediated reshaping of the host mycobiome is multifactorial. Although TLPs possess broad β-1,3-glucanase activity, the in-planta community shifts observed in Fig. 4 are selective rather than a simple reflection of biochemical inhibition. Fungal taxa that can serve as potential food resources for PWNs become enriched, indicating that additional ecological processes modulate the outcome. Differential sensitivity among fungal guilds, nutrient redistribution caused by TLP-mediated glucan degradation, and competitive interactions within the fungal network likely amplify these selective shifts. The released simple sugars and oligosaccharides also alter nutrient availability, favoring metabolically flexible Ascomycota capable of rapid recolonization. Host-mediated effects may further contribute, as TLPs in plants can influence immunity and signaling^61^, and we cannot fully exclude weak host responses to the heterologous protein despite using heat-inactivated controls. Thus, the community restructuring triggered by BxylTLP6 arises from the combined effects of antifungal pressure, nutrient reallocation, microbial competition, and host responses, rather than from a one-to-one correspondence with in-vitro inhibition profiles.

A similar complexity is evident in the suppression of wood-decaying fungi by TLPs. PWNs do not preferentially feed on these fungi, yet TLPs are still capable of degrading their cell walls. Such degradation does not necessarily interfere with nematode foraging on fungal food sources, as PWNs engage in chemically mediated interactions with various organisms in their environment. In addition to the oligosaccharides shown in this study to attract PWNs, other attractants maybe guide nematode behavior. Intriguingly, wood-decaying fungi themselves have been reported to attract PWNs^125^, and nematophagous fungi *Esteya* spp. can also lure them^46^. Conversely, certain fungi may produce compounds that exert repellent effects on nematodes. Therefore, the reduced abundance of wood-decaying fungi in planta reflects ecological incompatibility rather than feeding preference, and whether PWNs actively avoid these fungi remains unclear. The chemical interactions between PWNs and distinct fungal guilds merit further investigation.

**Fig. 5:**
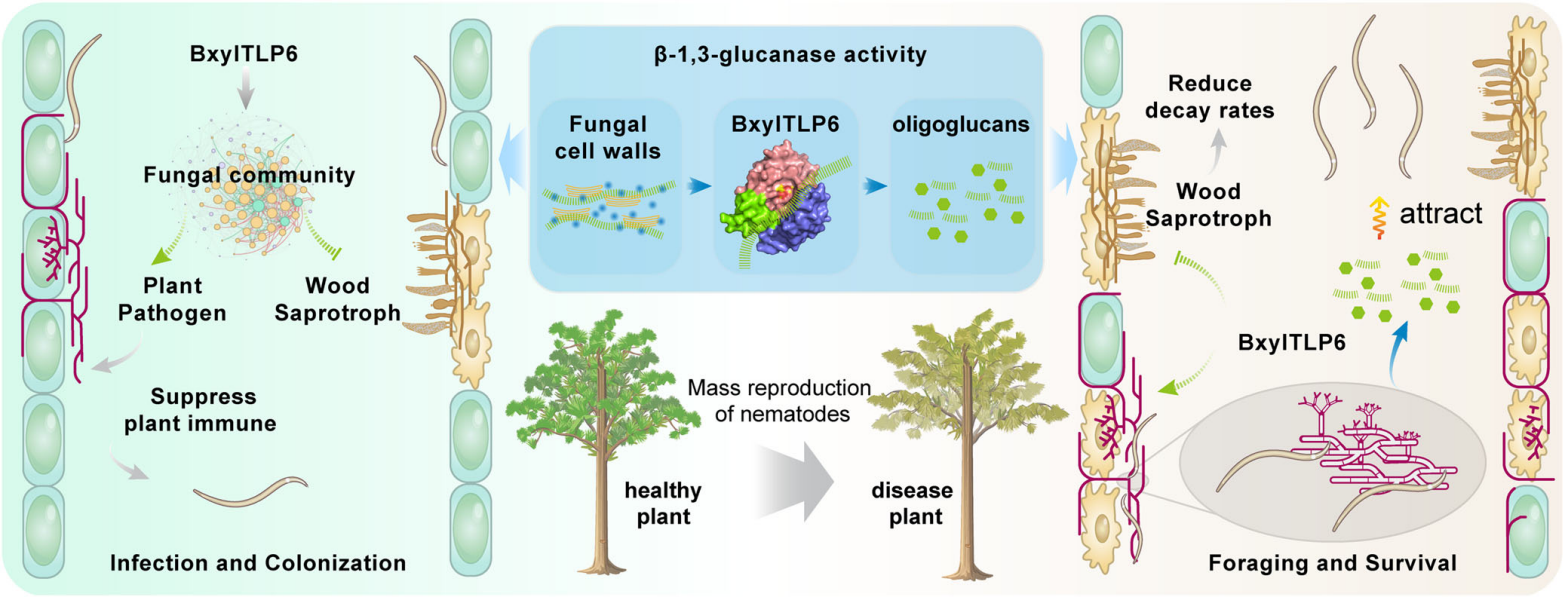
Schematic diagram illustrating the role of BxylTLP6. The central panel illustrates the protein function of BxylTLP6, which possesses β-1,3-glucanase activity that degrades fungal cell walls into oligoglucans. The left panel depicts the early stage of PWN infection in healthy pine trees, during which the nematodes primarily feed on plant cells because these resources are abundant and easier to access than fungi at this stage. The secreted BxylTLP6 helps modulate the endophytic fungal community by directly or indirectly increasing the abundance of pathogenic and parasitic fungi, which may assist PWNs in suppressing host defenses, while concurrently reducing wood-decaying fungi. As the nematodes proliferate and disease progresses, the pine gradually weakens and ultimately dies (right panel). After host death, PWNs shift to feeding on the fungal community. By suppressing wood-decaying fungi, BxylTLP6 may help limit tissue degradation and protect the nematode’s ecological niche. In addition, during nematode foraging, the oligoglucans released from BxylTLP6-mediated fungal cell-wall degradation may serve as attractive cues that guide PWNs toward fungal-rich microhabitats.

Taken together, we propose a conceptual model describing the possible role of BxylTLP6 in PWN infection and survival (Fig. 5). BxylTLP6 functions as a β-1,3-glucanase that degrades fungal cell walls into oligoglucans. During the early stage of PWN infection in healthy pine trees, the nematodes primarily feed on plant cells because these resources are abundant and easier to access than fungi. The secreted BxylTLP6 appears to modulate the endophytic fungal community by increasing the abundance of pathogenic and parasitic fungi, which may help PWNs suppress host defenses, while simultaneously reducing wood-decaying fungi. As the nematodes proliferate and disease progresses, the pine gradually weakens and eventually dies. After host death, PWNs shift to feeding on the fungal community. By suppressing wood-decaying fungi, BxylTLP6 may help reduce tissue degradation and protect the nematode’s ecological niche. In addition, the oligoglucans released through BxylTLP6-mediated fungal degradation may serve as foraging cues that attract PWNs to fungal-rich microhabitats.

Despite these insights, our study has several limitations. First, our analysis of the TLP gene family was not explicitly integrated with a comprehensive nematode phylogeny, which will be necessary to more rigorously link TLP diversification to nematode evolutionary history and lifestyle transitions. Second, the apparent association between TLP repertoire size and habitat microbial diversity is based on a limited set of species, and broader surveys across additional nematode species and environments are needed to test how general this pattern is. Third, although TLPs are β-1,3-glucanases that intrinsically degrade fungal cell walls and can influence host-associated microbial communities through at least three non-exclusive mechanisms, namely direct inhibition of sensitive fungi, the generation of cell wall–derived breakdown products that modify the local nutrient microenvironment, and modulation of host plant immunity that indirectly modulates the microbiota, these components were not dissected experimentally here; engineered TLP variants or mutants will be required to clarify their relative contributions. Fourth, although BxylTLP6 exhibits broad-spectrum antifungal activity, two fungal isolates in our system were insensitive to this effector, and the mechanisms underlying such insensitivity remain unknown. Finally, BxylTLP6 is probably only one of several antifungal or microbiome-modulating effectors secreted by PWN, and systematic identification of additional factors will be important to fully understand how nematodes modulate host-associated microbial communities.

## Supplementary information

Supplementary Information

Supplementary Data 1

Supplementary Data 2

Supplementary Data 3

Supplementary Data 4

## Data Availability

All data generated in this study have been deposited in Figshare and are available under the DOI: 10.6084/m9.figshare.30745067.

## Code availability

No custom code was used to generate or analyze the data in this study.
