# Supplementary material for: An antifungal effector from a plant-parasitic nematode modulates host fungal community composition and supports ecological fitness
Source: NPJ Biofilms Microbiomes. 2026 Mar 10;12:85. doi: 10.1038/s41522-026-00954-4 (PMC13106811; doi:10.1038/s41522-026-00954-4)
Supplement: Supplementary file 1 — Supplementary Information [file 41522_2026_954_MOESM1_ESM.pdf]

Supplementary information for

An antifungal effector from a plant-parasitic nematode modulates host fungal community composition and supports ecological fitness

Dong-Zhen Li[1,2], Yongxia Li[1,2*], Xuan Wang[1,2], Yuqian Feng[1,2], Yuhang Liu[1,2], Can Yang[1,2], Wei Zhang[1,2], Xiaojian Wen[1,2], Zhenkai Liu[1,2], Wandong Yin[1,2] and Xingyao Zhang[1,2]

[1] Key Laboratory of Forest Protection of National Forestry and Grassland Administration, Ecology and Nature Conservation Institute, Chinese Academy of Forestry, Beijing 100091, China
[2] Co-Innovation Center for Sustainable Forestry in Southern China, Nanjing Forestry University, Nanjing 210037, China
* Corresponding author**:** Yongxia Li (liyongxiaxjs@163.com)

Legends

Supplementary Figure 1. Secondary structure comparison of BxylTLP5 and BxylTLP6 with TLPs from the Protein Data Bank (PDB). Multiple sequence alignment of BxylTLP5 and BxylTLP6 with representative PDB TLPs (PDB IDs shown in the figure). Conserved residues are shaded by similarity. Secondary structure elements are displayed above the alignment for structural comparison.

Supplementary Figure 2. SDS-PAGE analysis of purified recombinant BxylTLP5 and BxylTLP6. Lane 1, uninduced sample; lane 2, protein molecular weight marker; lane 3, purified recombinant BxylTLP5; lane 4, purified recombinant BxylTLP6. Major bands migrate at approximately 27-30 kDa, consistent with the predicted molecular masses of BxylTLP5 (27.4 kDa) and BxylTLP6 (30.3 kDa).

Supplementary Figure 3. Standard curve for β-1,3-glucanase activity assays of BxylTLP5 and BxylTLP6. The red line represents the standard curve. The blue dashed line and the green dashed line represent the absorbance values of BxylTLP5 and BxylTLP6, respectively.

Supplementary Figure 4. RNAi silencing efficiency in PWN. (A) Uptake of solution by nematodes after 36 h soaking treatment, with FITC used as a fluorescent tracer. Strong fluorescence from the stylet to the entire intestine was observed after 36 h, indicating uptake of solution components during soaking. (B) Relative expression levels of *Bxyltlp5* and *Bxyltlp6* after 36 h soaking in dsRNA solution. (C) Relative expression levels of *Bxyltlp5* and *Bxyltlp6* in RNAi-treated nematodes after 7 days of

feeding on *B. cinerea* plates. Each dot represents an independent biological replicate (n = 5). Bars show mean ± SD. Different letters indicate significant differences (one-way ANOVA with Tukey's post hoc test, P < 0.05).

Supplementary Figure 5. Effects of RNAi targeting *Bxyltlp5* and *Bxyltlp6* on survival and body length of nematodes feeding on *B. cinerea* plates. RNAi-treated nematodes (dsGFP, dsBxyltlp5, dsBxyltlp6, dsBxyltlp5&6) were cultured on *B. cinerea* plates for 7 days. Each dot denotes an independent biological replicate (n = 20). Bars show mean ± SD. Statistical comparisons among groups were performed using one-way ANOVA (P < 0.05 considered significant).

Supplementary Figure 6. Chemotaxis assays of *B. xylophilus*. (A) Schematic diagram showing β-1,3-glucanase-mediated degradation of fungal cell walls. (B) Diagram of the chemotaxis assay setup. (C) Chemotactic responses of PWN to D-glucose, laminaripentaose, or fructose. Bars show mean ± SD (n = 5 independent biological replicates). "Two asterisks" indicate P < 0.01 versus buffer control (unpaired t-test).

Supplementary Figure 7. Relative expression levels of *Bxyltlp5* and *Bxyltlp6* in RNAi-treated nematodes inoculated into seedlings after 7 days. Dots represent independent biological replicates (n = 5), bars show mean ± SD. Different letters indicate significant differences (one-way ANOVA with Tukey's post hoc test, P < 0.05).

Supplementary Figure 8. Migration, survival, and development of RNAi-treated nematodes targeting *Bxyltlp5* and *Bxyltlp6* inside seedlings. (A) Numbers of nematodes recovered from stem segments 3 cm and 5 cm above the inoculation site. (B) Total nematode numbers recovered per seedling. (C) Body length of recovered nematodes. Each dot indicates an independent biological replicate (n = 10). Bars show mean ± SD. Group comparisons were performed using one-way ANOVA with Tukey's post hoc test (P < 0.05).

Supplementary Figure 9. Venn diagram of fungal community OTUs in seedlings under different treatments.

Supplementary Figure 10. Community barplot analysis of microbial assemblages on phylum level under different treatments.

Supplementary Figure 11. Effects of different treatments on the relative abundance of Ascomycota and Basidiomycota. The data are presented as means ± SEM. Comparisons were carried out using one-way ANOVA followed by Tukey's test. Different letters indicate significant differences (P < 0.05).

Supplementary Figure 12. Co-occurrence networks of endophytic fungal community OTUs in the seedlings under different treatments. Red edges indicate positive correlations. Green edges indicate negative correlations.

Supplementary Figure 13. Variations in the composition of fungal functional groups inferred by FUNGuild.

Supplementary Figure 14. Antifungal activity of BxylTLP6 against ten fungal strains. Each strain includes two treatments: the top panels show the heat-inactivated BxylTLP6 control, and the bottom panels show the treatment with active BxylTLP6.

Supplementary Figure 15. Growth of PWN on ten fungal strains. (A) Observations of feeding behavior on PDA plates inoculated with different fungi. (B) Reproductive performance of nematodes on PDA plates with different fungi.

Supplementary Table 1. Primers used in this study.

Supplementary Data 1. List of genomic information used in this study.

Supplementary Data 2. Amino acid sequence information of TLPs.

Supplementary Data 3. Nutritional sources and habitats of different nematodes.

Supplementary Data 4. OTU Taxon Analysis.

Supplementary Figure 1

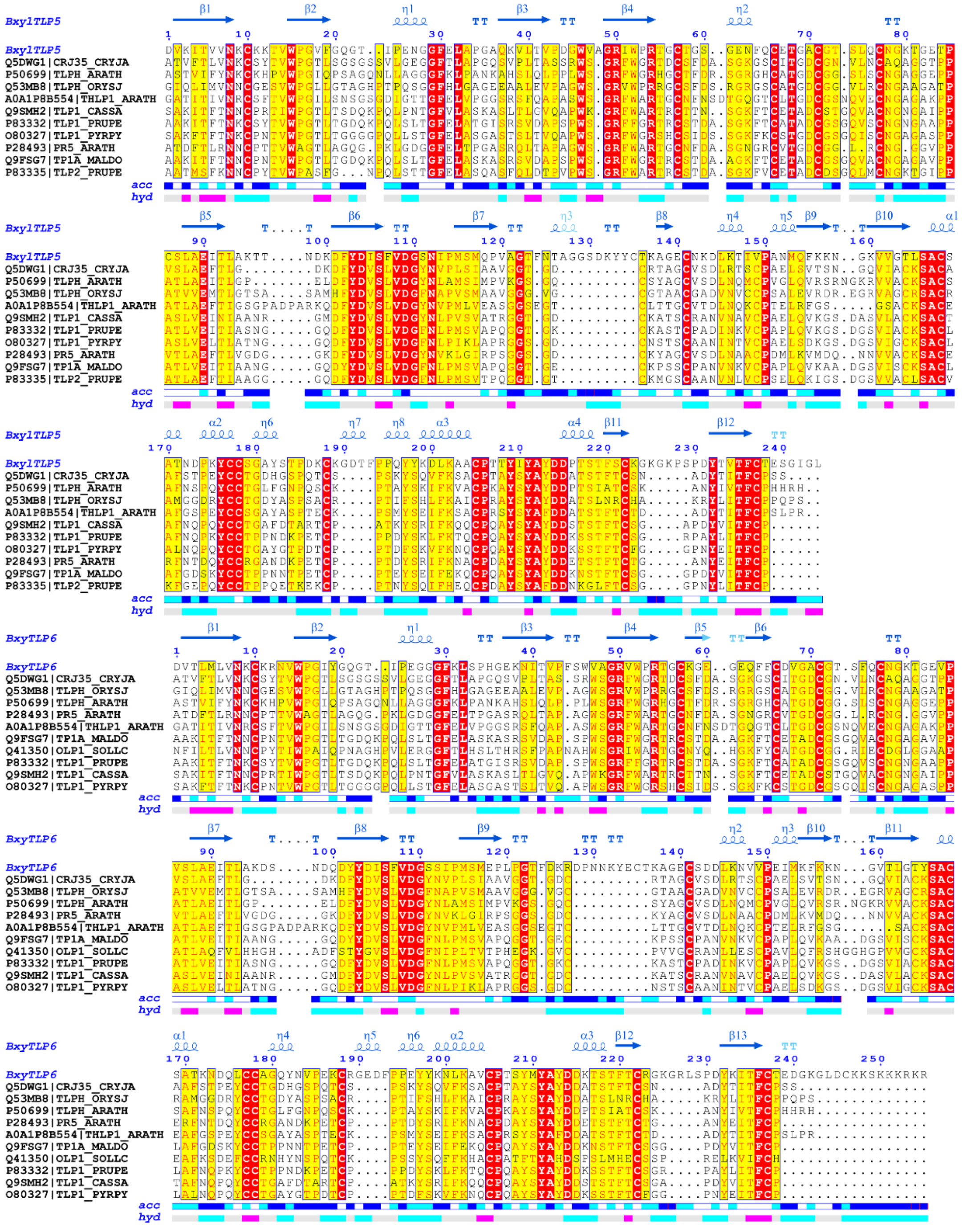

Supplementary Figure 2

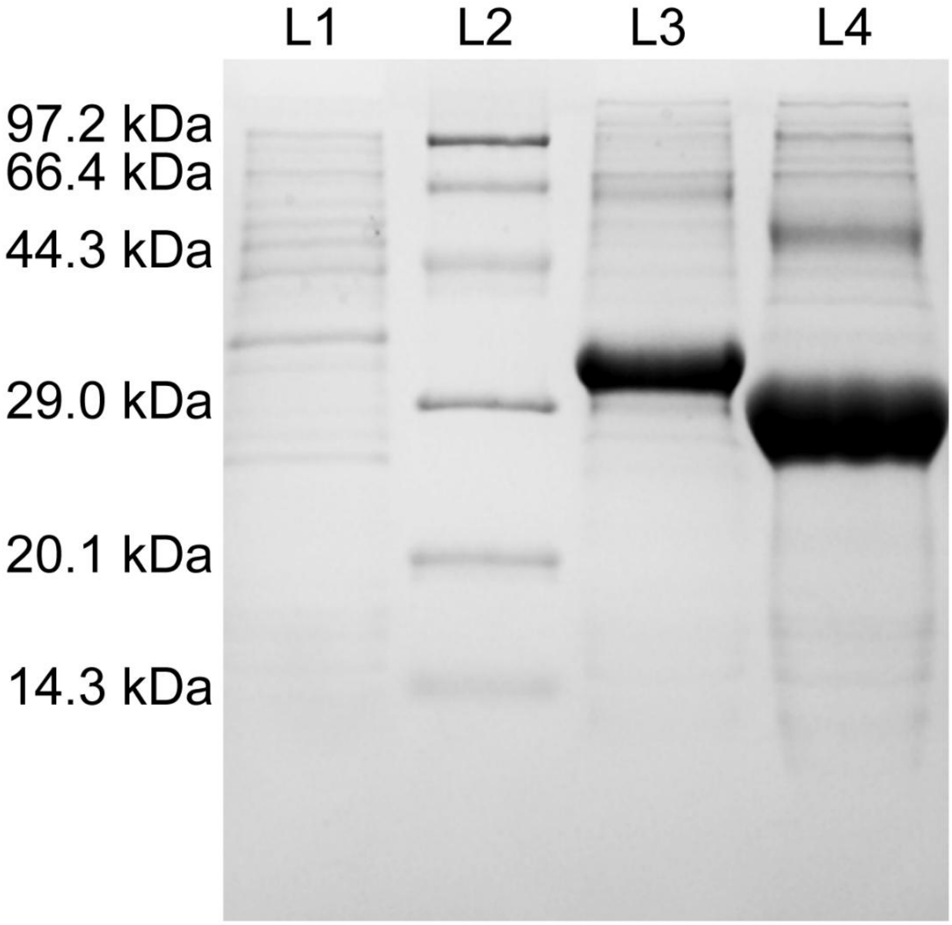

Supplementary Figure 3

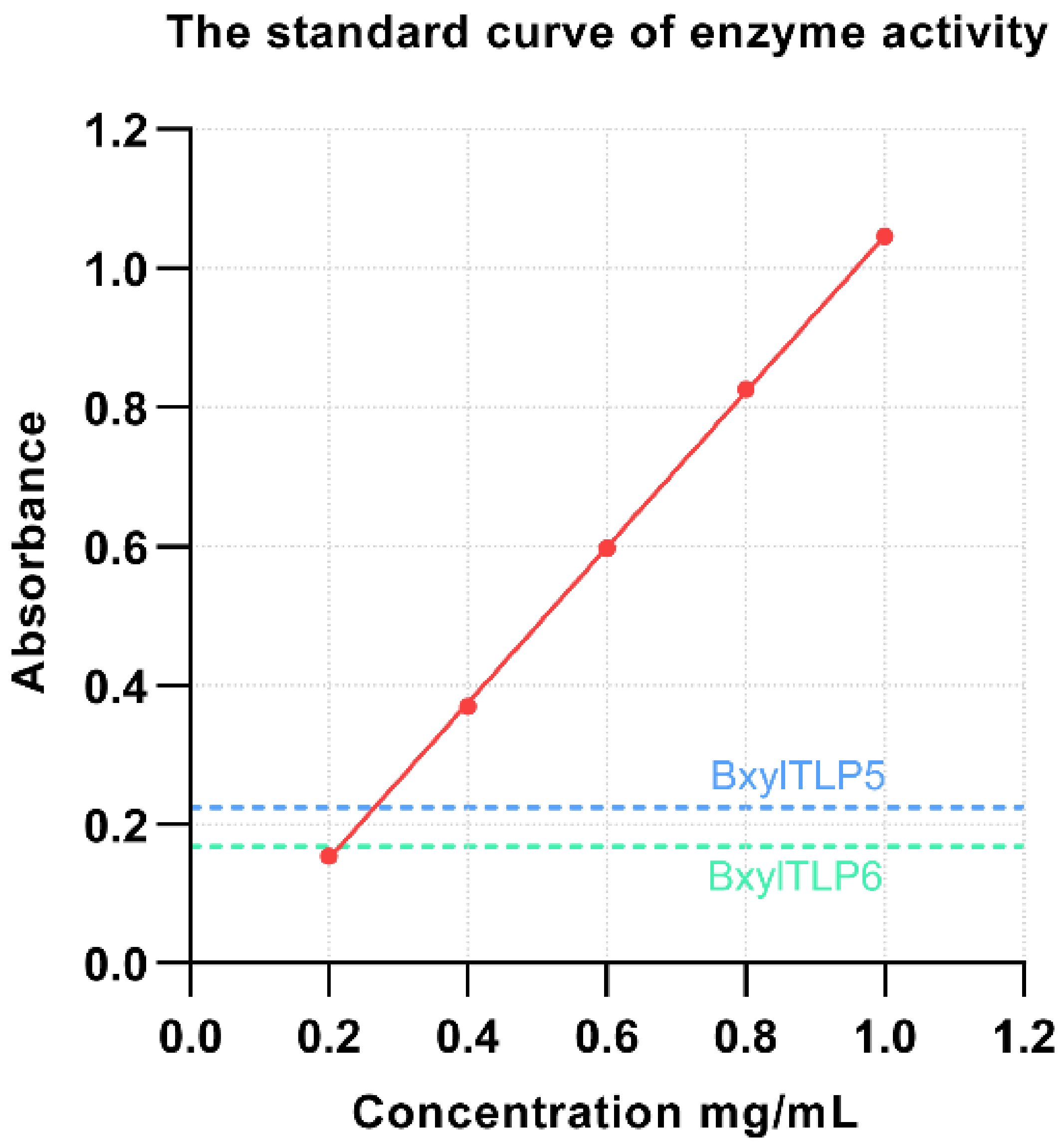

Supplementary Figure 4

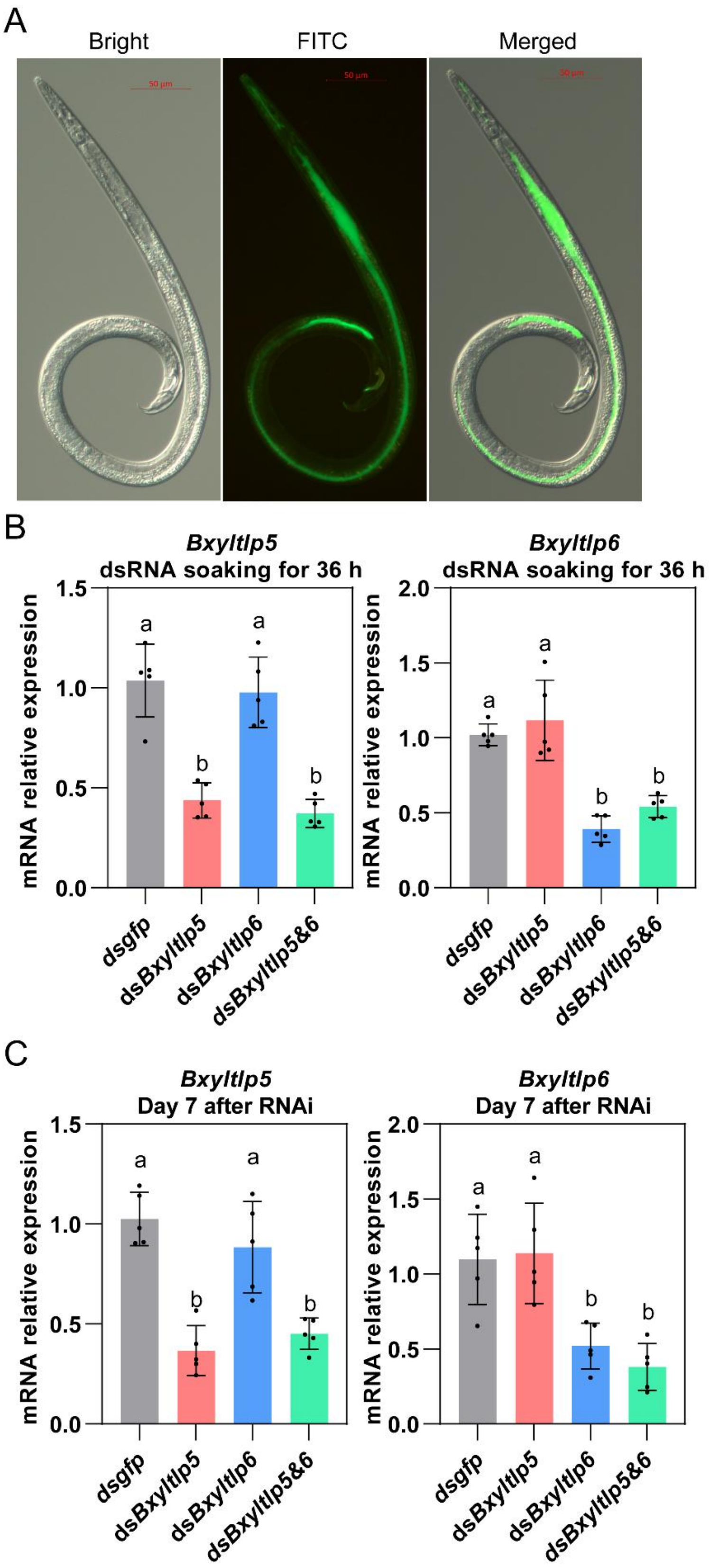

Supplementary Figure 5

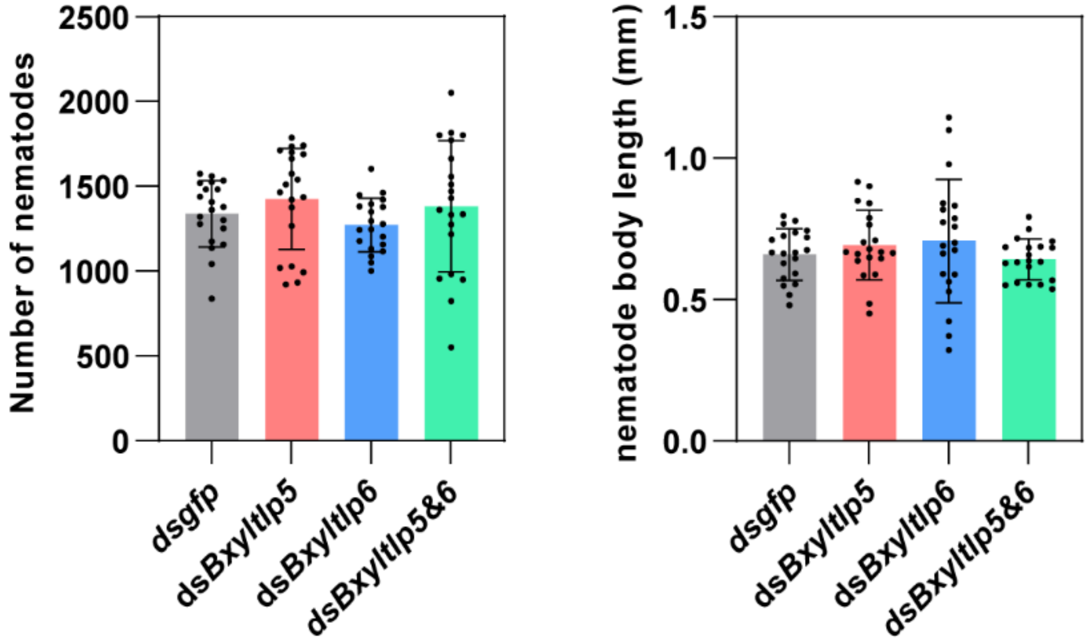

Supplementary Figure 6

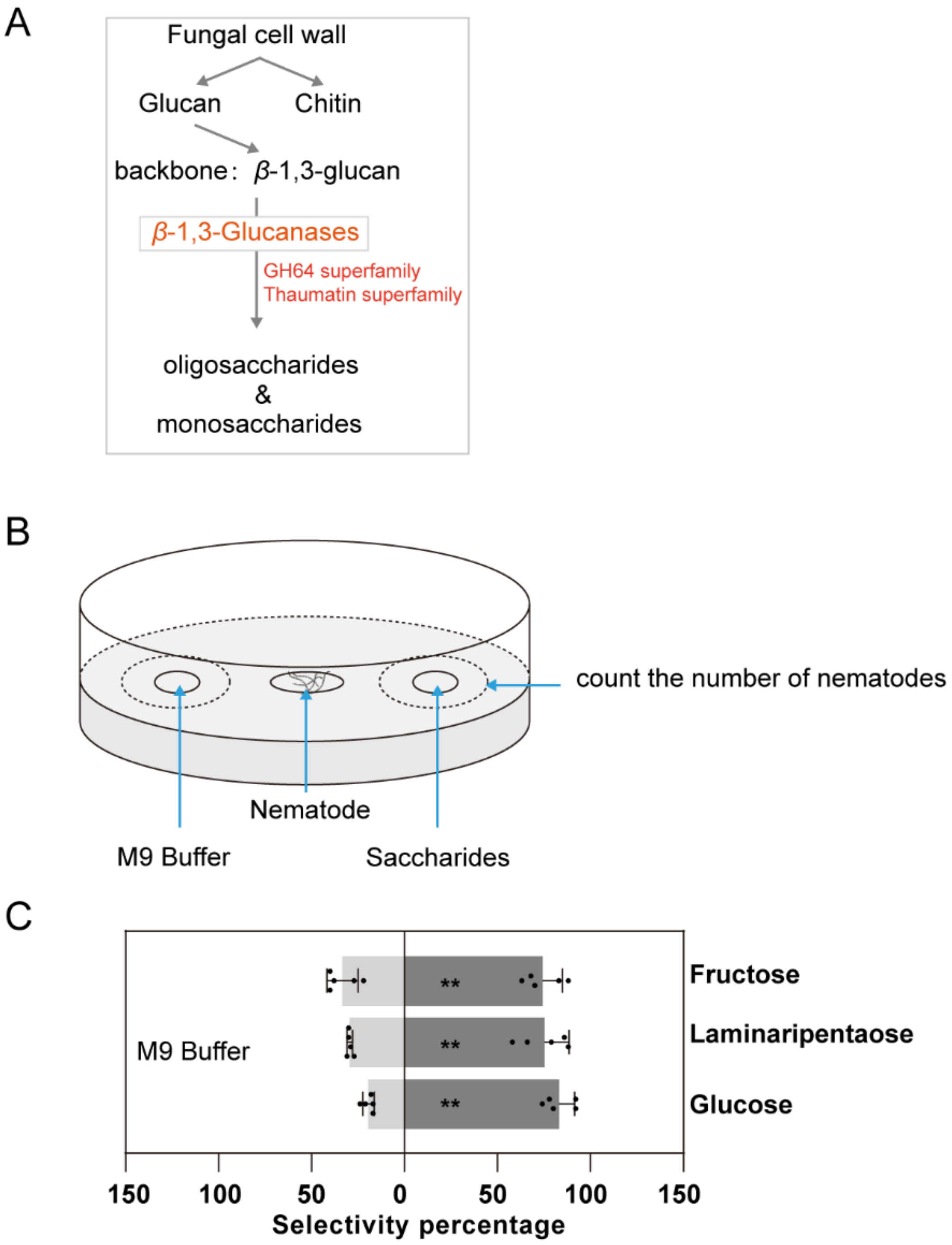

Supplementary Figure 7

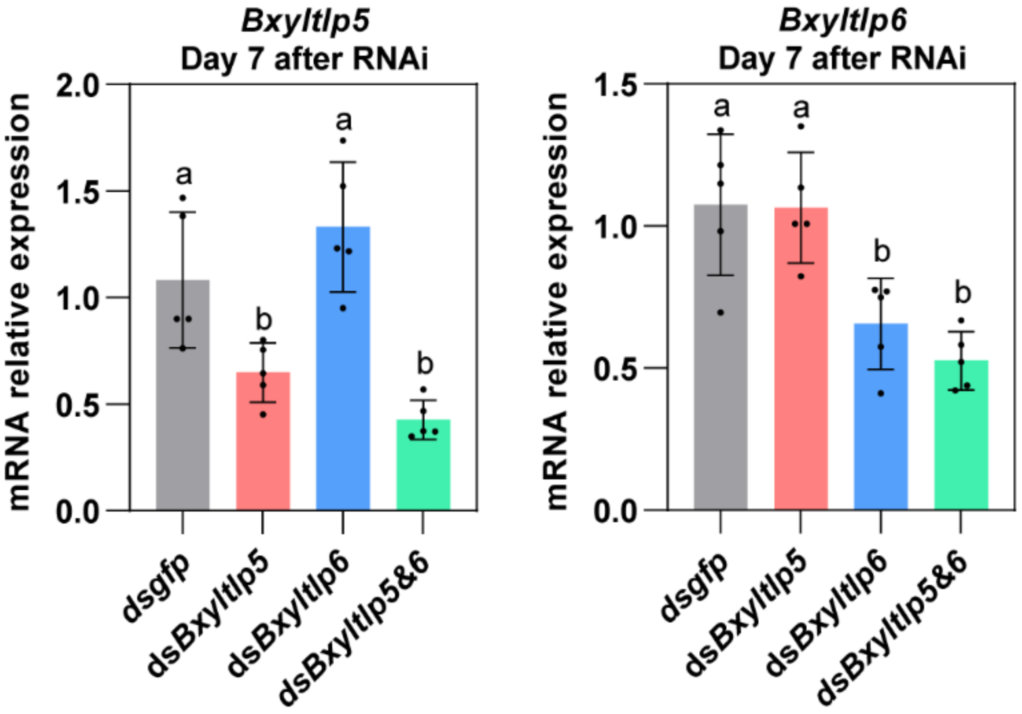

Supplementary Figure 8

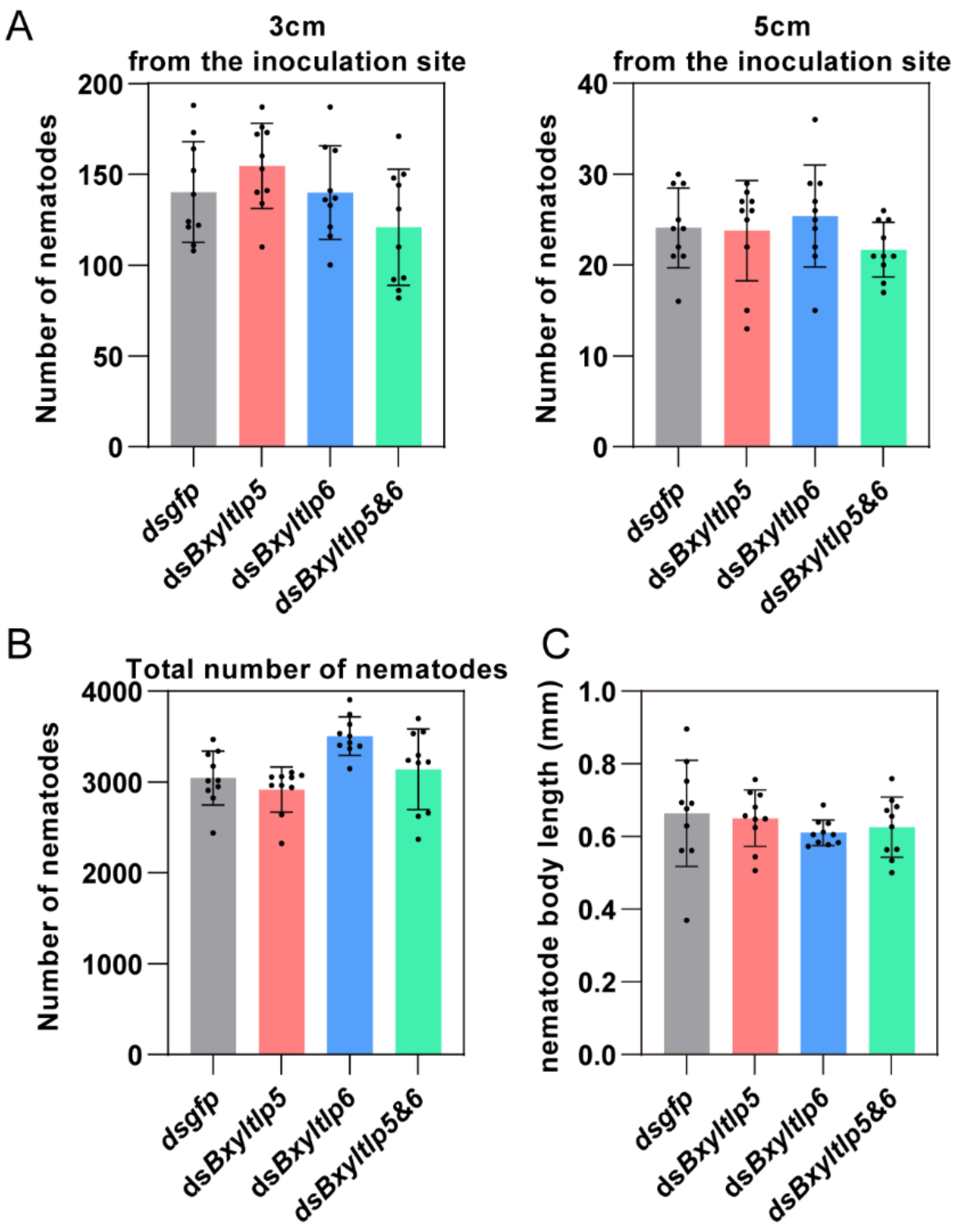

Supplementary Figure 9

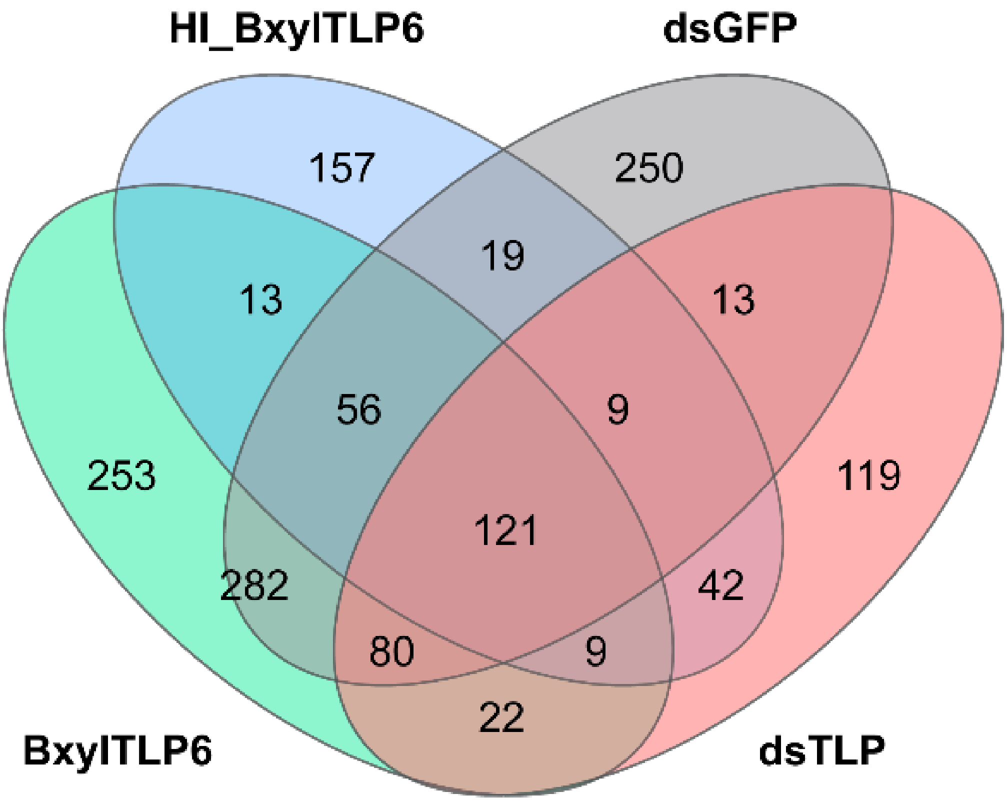

Supplementary Figure 10

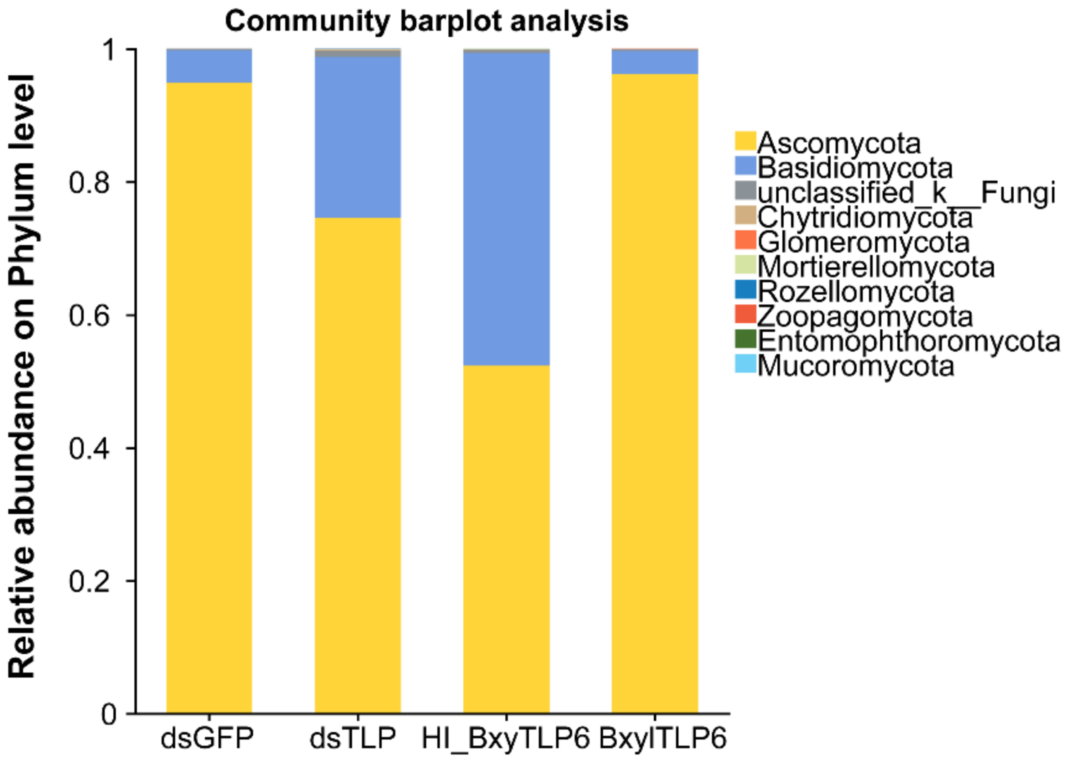

Supplementary Figure 11

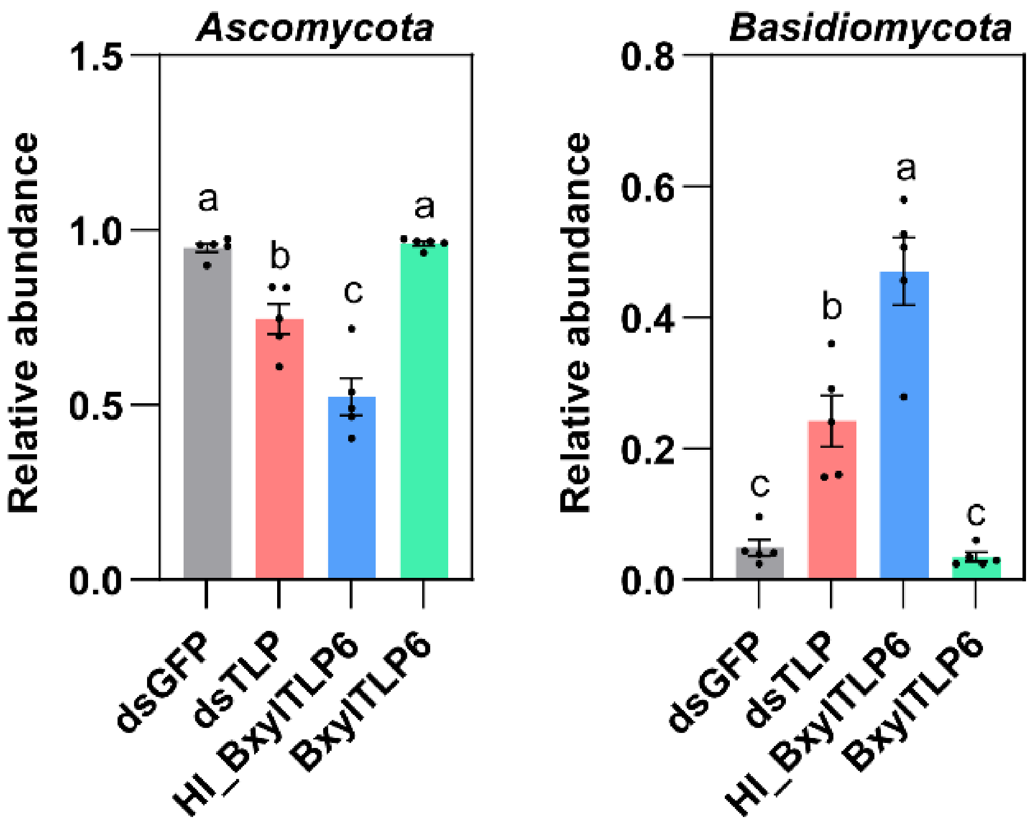

Supplementary Figure 12

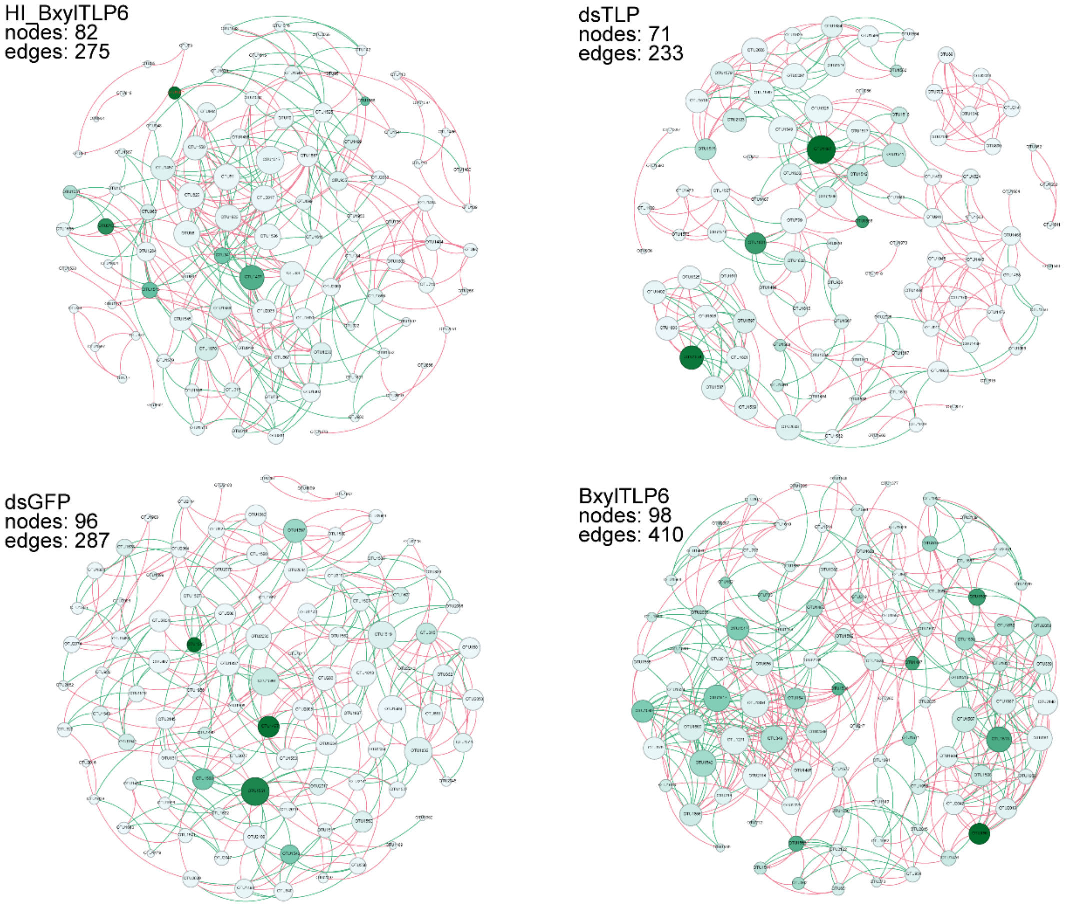

Supplementary Figure 13

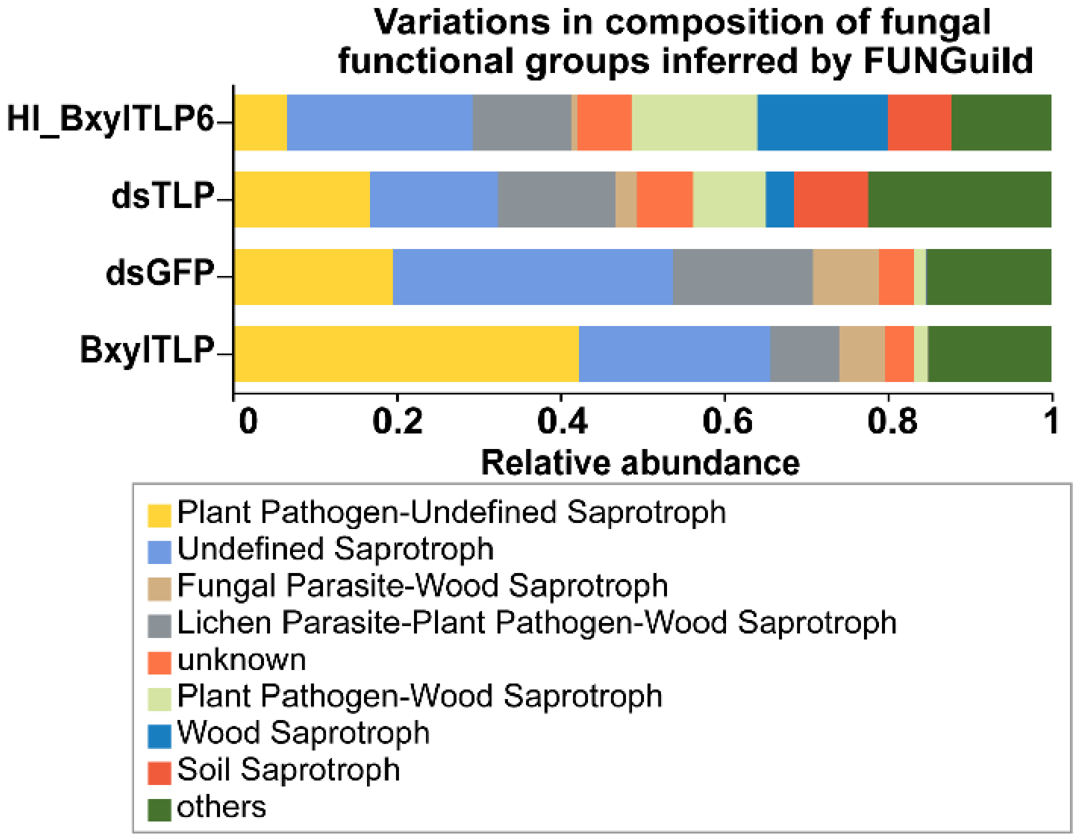

Supplementary Figure 14

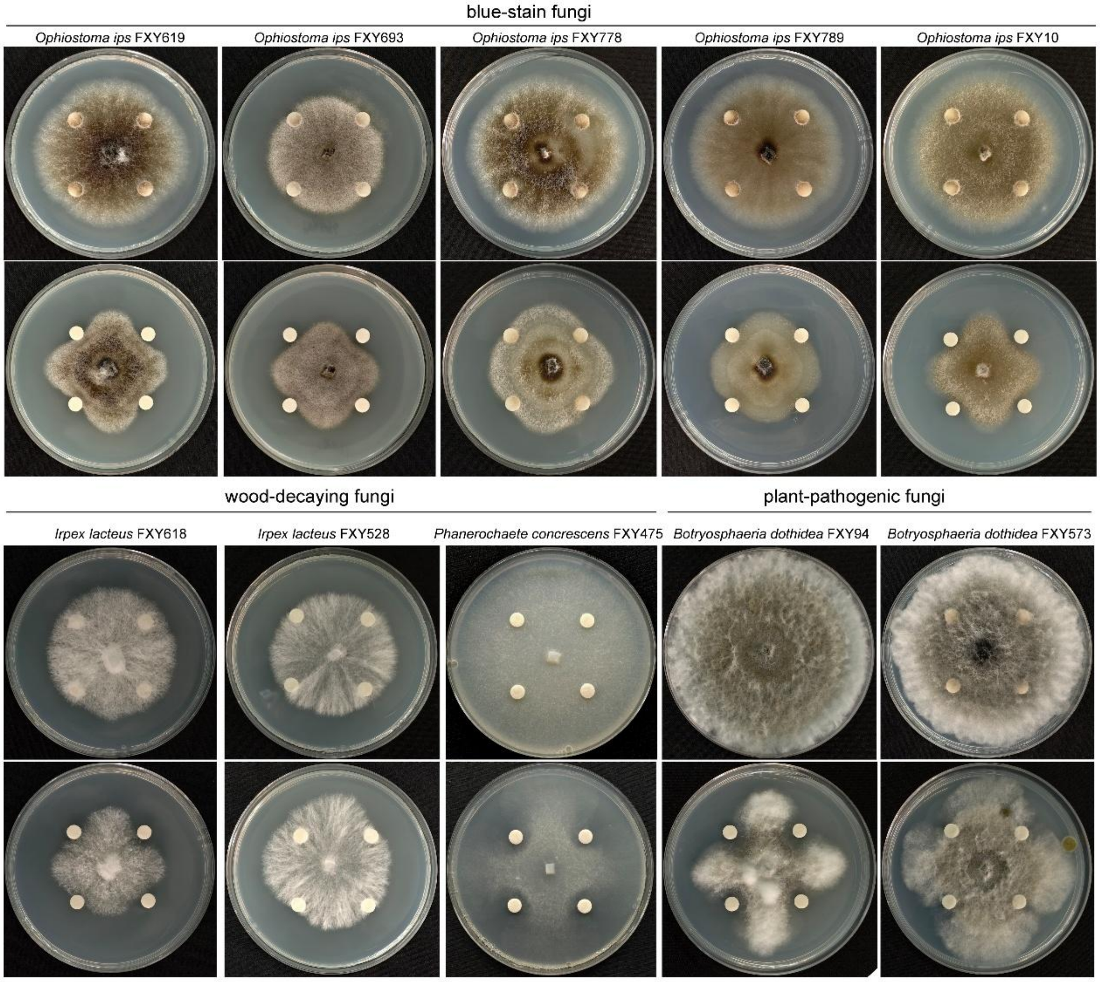

Supplementary Figure 15

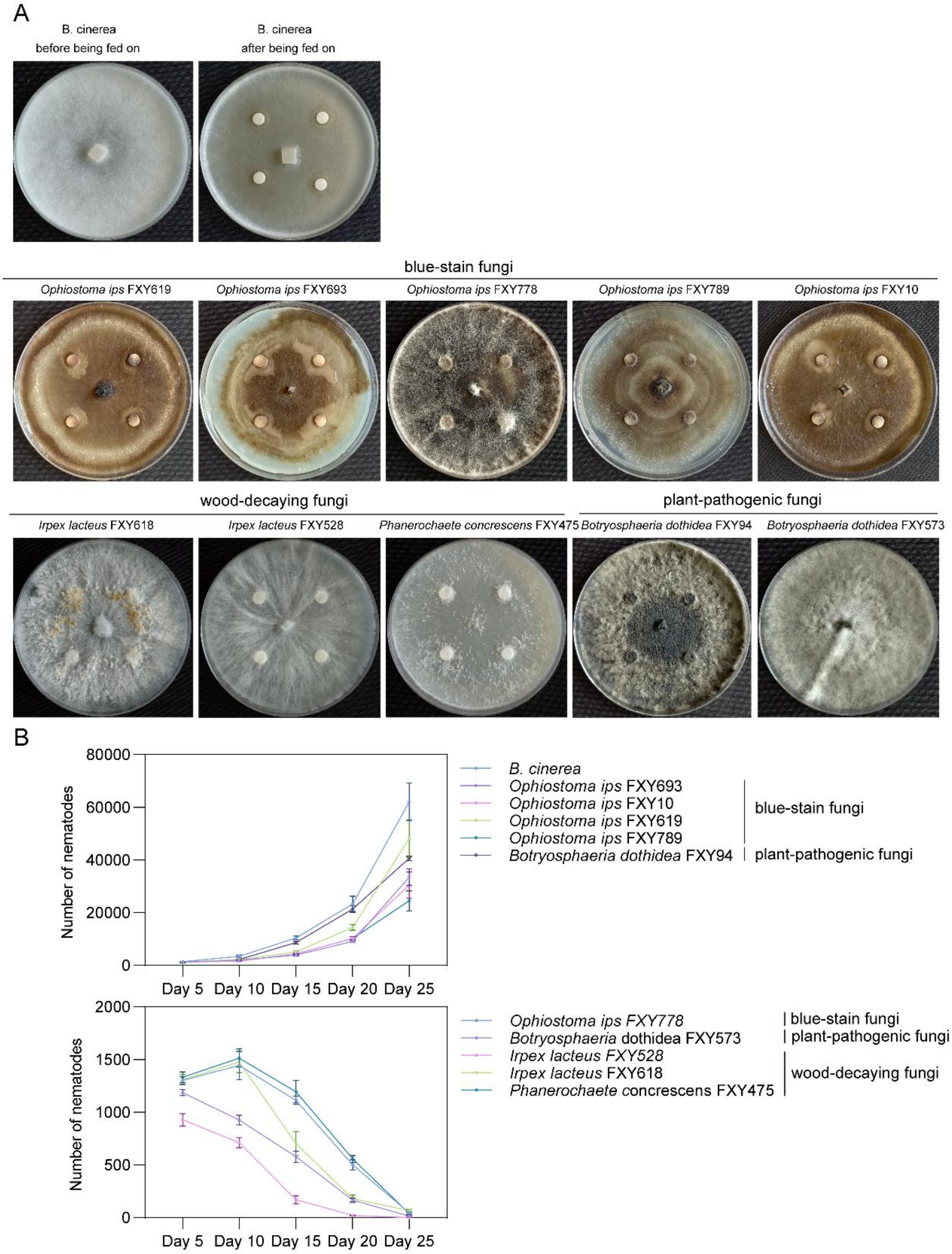

Supplementary Table 1. Primers used in this study.

Primer name	Primer sequence (5'-3')	Function
Bxyltlp5-F	ATGAACAAACTCGTCT	gene clone
Bxyltlp5-R	TTACAATCCGATGCCACT	gene clone
Bxyltlp6-F	ATGACAAACTCTGCAA	gene clone
Bxyltlp6-R	TTATCGTTTTCTCTTCTT	gene clone
qBxyltlp5-F	GCCAAGACCACCAATGACAA	RT-qPCR
qBxyltlp5-R	CGACAACCTTACCGTTCTTCTT	RT-qPCR
qBxyltlp6-F	GCAGCGACGACCTGAAGAA	RT-qPCR
qBxyltlp6-R	GCGGCAAGTAAATGTGGATGT	RT-qPCR
qActin-F	TCCGTACCCTGAAGTTGGCTAACC	RT-qPCR
qActin-R	AAGTGGAGACGAGGGAATGGAACC	RT-qPCR
dsBxyltlp5-T7-F	TAATACGACTCACTATAGGTAAGGTTGTCGGCACTCTGT	dsRNA synthesis
dsBxyltlp5-R	ACAATCCGATGCCACTTTCA	dsRNA synthesis
dsBxyltlp5-F	TAAGGTTGTCGGCACTCTGT	dsRNA synthesis
dsBxyltlp5-T7-R	TAATACGACTCACTATAGGACAATCCGATGCCACTTTCA	dsRNA synthesis
dsBxyltlp6-T7-F	TAATACGACTCACTATAGGCCACAAAAAACGACCAGCTT	dsRNA synthesis
dsBxyltlp6-R	ACAATCCAATCCCTTGCCAT	dsRNA synthesis
dsBxyltlp6-F	CCACAAAAAACGACCAGCTT	dsRNA synthesis
dsBxyltlp6-T7-R	TAATACGACTCACTATAGGACAATCCAATCCCTTGCCAT	dsRNA synthesis
dsGFP-T7-F	TAATACGACTCACTATAGGGATGGTCCCAATTCTCGTGGAAC	dsRNA synthesis
dsGFP-R	CTTGAAGTTGACCTTGATGCC	dsRNA synthesis
dsGFP-F	TGGTCCCAATTCTCGTGGAAC	dsRNA synthesis
dsGFP-T7-R	TAATACGACTCACTATAGGGACTTGAAGTTGACCTTGATGCC	dsRNA synthesis
ITS1F	CTTGGTCATTTAGAGGAAGTAA	ITS sequencing

ITS2R	GCTGCGTTCTTCATCGATGC	ITS sequencing
DIGBxyltlp5-F	TTCGTCGATGGCTCCAACAT	anti-sense probe synthesis
DIGBxyltlp5-R	TCGTTGGTAGCGGAGCAAG	sense probe synthesis
DIGBxyltlp6-F	AATGGGGTTACTCTGGGCAC	anti-sense probe synthesis
DIGBxyltlp6-R	CCGCGGCAAGTAAATGTGG	sense probe synthesis
